# Parallel single-cell and bulk transcriptome analyses reveal key features of the gastric tumor microenvironment

**DOI:** 10.1186/s13059-022-02828-2

**Published:** 2022-12-22

**Authors:** Boxi Kang, Jordi Camps, Biao Fan, Hongpeng Jiang, Mahmoud M. Ibrahim, Xueda Hu, Shishang Qin, Dennis Kirchhoff, Derek Y. Chiang, Shan Wang, Yingjiang Ye, Zhanlong Shen, Zhaode Bu, Zemin Zhang, Helge G. Roider

**Affiliations:** 1 BIOPIC, Beijing Advanced Innovation Centre for Genomics, and School of Life Sciences, Peking University, Beijing, China; 2 Biomedical Data Science, Research & Early Development Oncology, Bayer AG, Berlin, Germany; 3 Department of Gastrointestinal Surgery, Key Laboratory of Carcinogenesis and Translational Research (Ministry of Education), Peking University Cancer Hospital and Institute, Beijing, China; 4 Department of General Surgery, Beijing Friendship Hospital, Capital Medical University, Beijing, China; 5 Department of Gastroenterological Surgery, Peking University People’s Hospital, Beijing, China; 6 Biomedical Data Science, Research & Early Development preMed, Bayer AG, Wuppertal, Germany; 7 Immuno Oncology, Research & Early Development Oncology, Bayer AG, Berlin, Germany; 8 Biomedical Data Science, Research & Early Development Oncology, Bayer US, Cambridge, MA USA; 9 Laboratory of Surgical Oncology, Peking University People’s Hospital, Beijing, China; 10 Beijing Key Laboratory of Colorectal Cancer Diagnosis and Treatment Research, Beijing, China; 11 Peking-Tsinghua Centre for Life Sciences, Academy for Advanced Interdisciplinary Studies, Peking University, Beijing, China; 12 Oncology Precision Medicine, Research & Early Development Oncology, Bayer AG, Berlin, Germany

**Keywords:** Tumor microenvironment, Gastric cancer, Single-cell RNA transcriptomics

## Abstract

**Background:**

The tumor microenvironment (TME) has been shown to strongly influence treatment outcome for cancer patients in various indications and to influence the overall survival. However, the cells forming the TME in gastric cancer have not been extensively characterized.

**Results:**

We combine bulk and single-cell RNA sequencing from tumors and matched normal tissue of 24 treatment-naïve GC patients to better understand which cell types and transcriptional programs are associated with malignant transformation of the stomach. Clustering 96,623 cells of non-epithelial origin reveals 81 well-defined TME cell types. We find that activated fibroblasts and endothelial cells are most prominently overrepresented in tumors. Intercellular network reconstruction and survival analysis of an independent cohort imply the importance of these cell types together with immunosuppressive myeloid cell subsets and regulatory T cells in establishing an immunosuppressive microenvironment that correlates with worsened prognosis and lack of response in anti-PD1-treated patients. In contrast, we find a subset of IFNγ activated T cells and HLA-II expressing macrophages that are linked to treatment response and increased overall survival.

**Conclusions:**

Our gastric cancer single-cell TME compendium together with the matched bulk transcriptome data provides a unique resource for the identification of new potential biomarkers for patient stratification. This study helps further to elucidate the mechanism of gastric cancer and provides insights for therapy.

**Supplementary Information:**

The online version contains supplementary material available at 10.1186/s13059-022-02828-2.

## Background

Gastric cancer (GC) is the fifth most common malignancy and the third-leading cause of cancer-related mortality worldwide [1]. Despite a gradual decrease in incidence, the global burden remains high, especially in certain regions such as Asia and Latin America [2]. Although early detected gastric cancer responds well to treatment, advanced gastric cancer tends to be an aggressive disease with median survival times of only 9–10 months [3]. Molecular profiling of gastric cancers yielded several sub-classes, including Epstein–Barr virus (EBV)-positive, microsatellite instable (MSI), genomically stable (GS), or chromosomal instable (CIN) [4, 5]. Immune checkpoint inhibition (ICI) therapy has shown promising results in metastatic gastric cancer patients with EBV-positive or MSI tumors with both achieving nearly 100% overall response rate (ORR). In contrast, CIN and GS tumors only attained an ORR of 12 and 5%, respectively [6]. Aside from tumor cells, immune cells and fibroblasts in the tumor microenvironment have been shown to affect the efficacy of cancer immunotherapy [7]. It is therefore important to stratify the baseline cellular milieu of the stomach to clarify the composition and property of tumor-infiltrating immune cells and stromal plasticity in gastric cancer.

The tumor microenvironment (TME) largely composed of lymphocytes, myeloid cells, endothelial cells, and cancer-associated fibroblasts is known for its contribution to inflammation, cancer immune suppression, angiogenesis, and metastasis [8, 9]. In gastric cancer, stromal cell signatures have been associated with worsened patient survival along with therapy resistance [10, 11] and promote tumor invasion through activating matrix remodeling, immune crosstalk, metabolic effects, and soluble secreted factors [9, 12]. However, the cell types contributing to these malignant characteristics of the gastric TME and their abundance in normal and tumor tissue are still poorly understood.

Applying single-cell RNA-seq has been very successful in characterizing the TME of other cancer indications at high resolution and was used recently to characterize gastric cancer cells [13–15]. Here we used scRNA-seq to comprehensively profile the TME of tumor and matched normal samples from 24 gastric cancer patients to generate a high-resolution cell atlas of the gastric tumor stroma. By combining this single-cell data with matched bulk RNA-seq data, we captured important deregulated biological processes and identified the cell types associated with poor survival and resistance to anti-PD1 treatment in gastric cancer.

## Results

### Global cellular microenvironment landscape in gastric cancer

**Fig. 1:**
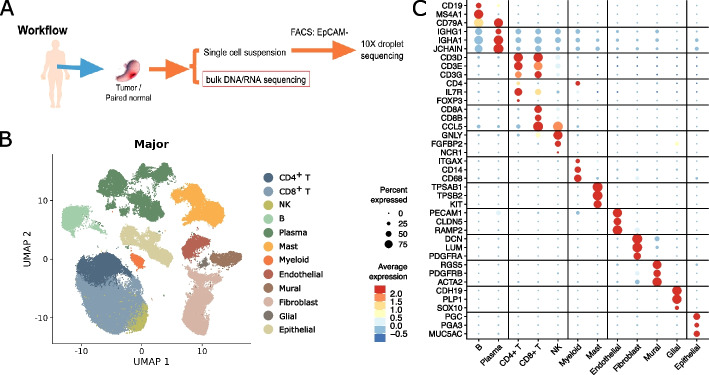
Cellular landscape of EPCAM-negative cells in non-malignant and malignant gastric patient samples. **A** Study overview: matched malignant and non-malignant stomach samples were obtained from a total of 24 patients. Samples were processed in parallel as bulk specimen through RNA and exome-sequencing and single cells through droplet RNA sequencing after depleting of EPCAM-positive cells. **B** UMAP of 96,623 cells, color coded for major cell type. **C** Dotplot showing the scaled average expression together with the percentage of expression of marker genes per major cell type

To characterize the TME in gastric cancer, we performed bulk tissue RNA sequencing (RNA-seq) and whole exome-sequencing together with single-cell RNA-seq (scRNA-seq) of tumor and matched non-malignant gastric tissue samples from 24 treatment-naive gastric cancer patients (Fig. 1a, Additional file 1: S1a and Additional file 2: Table S1). For identifying the cell types constituting the gastric TME, samples were rapidly digested into single cells, depleted for EPCAM-positive epithelial cells by fluorescent-activated cell sorting (FACS) to enrich for all cells of non-epithelial origin and analyzed using scRNA-seq. The resulting 96,623 cells were clustered into eleven major cell types and further into 81 subtypes (Fig. 1b, Additional file 1: Fig. S1f and “Methods”). The major clusters were annotated according to the expression of defining marker genes as either B cells (*CD19*, *MS4A1*), plasma cells (*IGHG1*, *CD79A*), CD4^+^ T cells (*CD3D*, *CD4*), CD8^+^ T cells (*CD3D*, *CD8A*), natural killer (NK) cells (*NCR1*, *FGFBP2*), myeloid cells (*CD14*, *CD68*), mast cells (*TPSAB1*, *TPSB2*), endothelial cells (*RAMP2*, *PECAM1*), fibroblasts (*DCN*, *LUM*), mural cells (*PDGFRB*, *ACTA2*), glial cells (*PLP1*, *SOX10*), or epithelial cells (*PGC*, *PGA3*) (Fig. 1c). To characterize the differences between the TME and normal gastric stroma in the following, we quantified the change in abundance and cellular activity associated with malignant transformation for each of the stromal cell types.

### Tumor stroma is characterized by the presence of activated fibroblasts

**Fig. 2:**
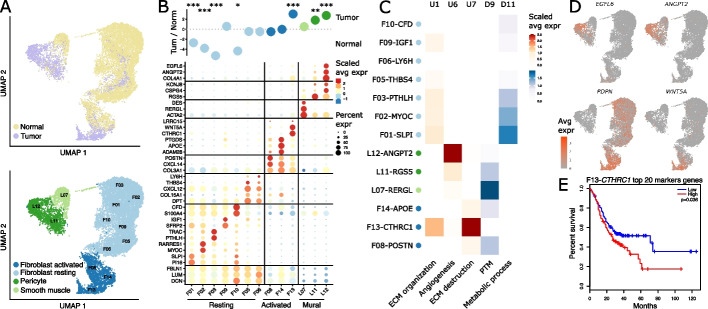
Transcriptional reprogramming in cancer-associated fibroblasts. **A** UMAP of 16,492 fibroblast cells color coded for tissue (top) and cluster annotation (bottom). **B** Log ratio of average fraction per fibroblast cluster in tumor to normal tissue (*n* = 20) (top). Wilcoxon rank-sum test with holm correction, *: *p* < 0.05, **: *p* < 0.01, ***: *p* < 0.001. Dotplot showing the scaled average expression and the percentage of expression of top markers per fibroblast cluster (bottom). **C** Scaled average expression of fibroblast implicated gene clusters (Additional file 1: Fig. S4). **D** Top marker genes for F12-*ANGPT2* and F13-*CTHRC1* connected to gene cluster U6 and U7 respectively. **E** Overall survival of gastric cancer patients in TCGA, groups split by the top 20 marker gene signature of F13-*CTHRC1*. Gene signatures U1, U6, and U7 reflect gene clusters that were upregulated in gastric tumor samples while gene signatures D9 and D11 reflect gene clusters that were downregulated in gastric tumor samples

To determine if any fibroblast and related mural cell subtypes are associated with malignant transformation, we performed re-clustering of the corresponding 16,492 cells in our dataset which resulted in 15 cell clusters (Fig. 2a). By evaluating the expression of specific marker genes across these clusters, we found mural cells to be clearly separated from fibroblasts and to be comprised of pericytes characterized for instance by the expression of *RGS5*, and smooth muscle cells expressing among other genes *ACTA2* and *DES* (Additional file 1: Fig. S2a). Fibroblasts on the other hand were distinctly divided into resting and activated cells as could be inferred from the expression of markers for resting fibroblasts such as *S100A4*, *CFD*, and *DPT* and markers for active cells like *POSTN*, *CXCL14*, and *COL3A1* [9, 16–18] (Fig. 2b, S2a). A closer look at the individual cell subtypes revealed multiple unique genes for each cluster, underlining the heterogeneity of both gastric fibroblast and mural cells (Fig. 2b). Applying consensus non-negative matrix factorization (cNMF) [19], we identified distinct gene expression programs for the different fibroblast subtypes confirming that the different clusters indeed represent either different cell subtypes or cell activation states (Additional file 1: Fig. S3a-b). The fibroblast subtypes further displayed different characteristics in extracellular matrix-related gene expression, pointing to possible distinct functions in shaping the extracellular matrix (Additional file 1: Fig. S3c-h). Cluster fraction comparison between malignant and non-malignant samples highlighted subtype F13-*CTHRC1* as highly cancer-associated fibroblasts (CAFs) while revealing a drastic reduction of several resting fibroblast clusters in the gastric TME (Fig. 2b and S2b). In line with this finding, expression of *CTHRC1* is upregulated in tumors from many different indications in the cancer genome atlas (TCGA) cohort (Additional file 1: Fig. S2c). Comparing subtype F13-*CTHRC1* to a recently published fibroblast compendium revealed a strong similarity of these cells to activated myofibroblasts specifically expressing for instance *COL3A1*, *ACTA2*, and *CTHRC1 *[18]. Diffusion map analysis between resting and activated fibroblasts positioned resting fibroblasts *F01-SLPI* and F13-*CTHRC1* at both ends of a trajectory (Additional file 1: Fig. S2d), pointing towards activated fibroblasts emerging from resting fibroblasts.

### Expression of CTHRC1-positive fibroblast-specific genes is linked to poor survival

In addition to changes in cell type frequencies, many molecular pathways have been described to be deregulated in gastric tumors compared to healthy normal tissue [20]. To better characterize transcriptional changes associated with malignant transformation, we integrated our bulk and single-cell RNA-seq data and identified upregulated (designated by capital U) and downregulated (designated by a capital D) gene clusters that harbor different expression patterns across the single-cell populations (“Methods” and Additional file 1: Fig. S4a). Multiple upregulated and downregulated gene clusters specifically expressed in individual fibroblast subsets were identified (Additional file 1: Fig. S4b-c). Among upregulated clusters, U1 was expressed in resting and activated fibroblasts and strongly enriched with genes associated with gene ontology (GO) terms linked to remodeling of the extracellular matrix. In addition, clusters U6 and U7 were highly expressed in L12-*ANGPT2* pericytes and F13-*CTHRC1* CAFs, respectively (Fig. 2c, S4c). Cluster U6 thereby contained genes like *ACAN* and *COL5A3* which are involved in extracellular matrix organization as well as genes like *ANGPT2*, *EGFL6*, *PDGFRB*, and *PGF* (Additional file 1: Fig. S4b, S4d) which are connected to growth factor responses and angiogenesis in the GO ontology. These genes have also been reported as highly expressed in multiple cancer types and as being associated with angiogenesis and tumor invasion [21]. Cluster U7 contained genes related to fibroblast proliferation (*WNT5A* and *WNT*2), matrix remodeling (*MMP1* and *MMP3)*, and cell migration (*PDPN* and *TWIST1)* (Fig. 2d, S4b,d). All genes from cluster U7 were specifically expressed in F13-*CTHRC1* activated fibroblasts and were associated with poor survival in an independent gastric cancer cohort (Fig. 2e) as well as in several cancer indications from TCGA (Additional file 1: Fig. S2e-f). This is in alignment with previous reports that implicated these genes in cancer progression and bad outcome [22–26].

### Tumor endothelial cells swap immune attraction for angiogenic pathways

**Fig. 3:**
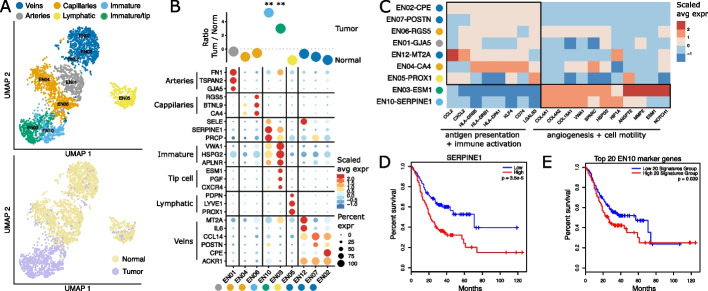
Hybrid endothelial cell states in gastric cancer. **A** UMAP of 3684 endothelial cells color coded for tissue (left) and cluster annotation (right). **B** Log ratio of average fraction per endothelial cell cluster in tumor to normal tissue (*n* = 20) (top). Wilcoxon rank-sum test with holm correction, **: *p* < 0.01. Dotplot of top markers per cluster showing the scaled average expression and the percentage of expression (bottom). **C** Heatmap of differentially expressed genes between endothelial cells of non-malignant and malignant biopsies contained in indicated gene ontology terms. Shown is the scaled average expression and the percentage of expression. **D, E** Overall survival of gastric cancer patients in TCGA, groups split by *SERPINE1 levels and top 20 marker genes of EN10 cluster, respectively.*

Endothelial cells are key components of the TME since supply of energy and nutrients through active blood circulation, rather than passive diffusion, is required for continuous tumor growth [27]. Upon analyzing the 3684 endothelial cells in our data, we found nine clusters that corresponded to six different endothelial cell subtypes (Fig. 3a). These endothelial clusters were associated with arteries (*GJA5, TSPAN2*), capillaries (*CA4, BTNL9*), immature endothelial cells (*HSPG2, VWA1*), tip cells (*PGF*), lymphatic cells (*PROX1, LYVE1*), and vein cells (*ACKR1*) which could be further delineated into postcapillary cells (*CPE*), activated (*POSTN*), and *IL6*-expressing cells (Fig. 3b). This observation is in line with a recently reported taxonomy of endothelial cells obtained from healthy human lung and lung carcinoma samples [28], demonstrating the consistent character of these vasculature-associated cell types across tissues. Cluster fraction comparison between normal and malignant gastric tissue identified clusters EN10-*SERPINE1* and EN03-*ESM1*, hereafter referred to as activated endothelial cells, as almost exclusively tumor-specific cell types (Fig. 3b, S2g).

To define the functionality of tumor endothelial cells, we first performed differential gene expression analysis between activated endothelial cells and the remaining endothelial cells (Additional file 4: Table S3). Subsequent gene set enrichment analysis performed on the significantly differentially expressed genes revealed an increase of angiogenic and cell motility response pathways together with a decrease of antigen-presenting and immune defense genes in both EN10-*SERPINE1* and EN03-*ESM1*, indicating that immune cell attraction through endothelial cells is reduced in gastric cancer (Fig. 3c). This confirms observations from other cancer indications that point to a decrease of antigen-presenting pathways in endothelial cells [29, 30] and could be a reason for the synergistic effect of anti-angiogenic therapy together with ICI [31].

In line with angiogenesis being critical for sustained tumor growth, we speculated that these cell types might be linked to poor survival and found that *SERPINE1* expression was associated with a drastically worsened survival in the independent gastric cancer cohort from TCGA (Fig. 3d). *SERPINE1* is a serine protease inhibitor that mainly functions as a regulator of cell adhesion and spreading and has been reported as upregulated in multiple other cancer indications such as head and neck squamous cell carcinoma, leading to a poor prognosis [32]. SERPINs have been described as important genes in cancer and vascular co-option and therefore play an important role in cancer progression leading to tumor metastasis [33]. We found a comparable difference in survival using the signature of the 20 most specific genes of the EN10-*SERPINE1* cluster (Fig. 3e). Together, these findings indicate that EN10-*SERPINE1* endothelial cells could be targeted to block the metastatic spread of gastric tumors.

### Macrophages display an inflammation dichotomy

**Fig. 4:**
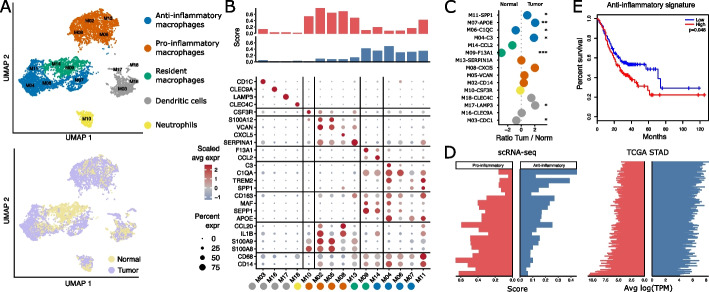
Pro- and anti-inflammatory macrophages are negatively correlated and highly diverse between gastric cancer patients. **A** UMAP of 10,646 myeloid cells color coded for tissue (bottom) and cluster annotation (top). **B** Bar plot of a proinflammatory (*IL1B*, *CCL20*, *S100A8*, *S100A9*) and anti-inflammatory (*CD163*, *MAF*, *SEPP1*, *APOE*) gene score per cell cluster (top). Dotplot of top markers per myeloid cluster showing the scaled average expression and the percentage of expression (bottom). **C** Log ratio of the average fraction per myeloid cluster annotation in tumor to normal tissue (*n* = 20). Wilcoxon rank-sum test with Holm correction, *: *p* < 0.05, **: *p* < 0.01, ***: *p* < 0.001. **D** Bar plot of the proinflammatory and anti-inflammatory gene signature from **B** in scRNA-seq data of our cohort (*n* = 20) and the TCGA-STAD cohort (*n* = 407). **E** Overall survival of gastric cancer patients in TCGA, groups split by expression level of the proinflammatory (left) and anti-inflammatory (right) gene signatures from B

To shed light on myeloid populations present in gastric tumors, we re-clustered the 10,546 myeloid cells in our dataset and identified fifteen subclusters which were subsequently determined to be either macrophages (*CD68*, *CD14*), dendritic cells (*FLT3*, *FCER1A*), or neutrophils (*CSF3R*) (Fig. 4a). The four dendritic cell clusters in our data matched dendritic cells found in hepatocellular and colorectal carcinoma [34, 35] and were annotated as conventional cDC1 M16-*CLEC9A*, cDC2 M03-*CD1C*, plasmacytoid M18-*CLEC4C*, and M17-*LAMP3* dendritic cells (Fig. 4b). Through differential gene expression analysis, macrophage clusters were identified as either proinflammatory (*S100A8*, *S100A9* [36], *IL1B* [37], and *CXCL8* [38]), anti-inflammatory (*APOE* [39], *MAF* [40], *C1QB*, and S*EPP1* [41]), or tissue-resident macrophages (*F13A1* [42] and *CCL2* [43]) (Fig. 4b). Tissue-resident macrophages were found to be distinct from anti-inflammatory macrophages through the expression of various genes including *FOLR2*, *CCL2*, *LYVE1*, *SEPP1*, and *F13A1*, all of which have been reported as markers of tissue-resident macrophages [44]. Myeloid cells were drastically increased in the TME with only tissue-resident macrophages higher in healthy stomach tissue. In particular, the gastric TME was characterized by a significant increase in M07-*APOE*, M11-*SPP1*, and M04-*C3* anti-inflammatory macrophages (Fig. 4c, S5).

Surprisingly, we found the expression of the above pro- and anti-inflammatory gene signature to be anti-correlated across patient samples in both our data as well as in the independent stomach adenocarcinoma cohort from TCGA (Fig. 4d). In line with a recent publication that linked poor survival to the presence of anti-inflammatory myeloid cells [45], the expression of our anti-inflammatory signature was significantly associated with reduced survival in the TCGA gastric cancer cohort (Fig. 4e). However, the abundance of the corresponding macrophage populations as determined by CIBERSORTx deconvolution of the bulk RNA-seq samples was not predictive for outcome (data not shown).

### Lymphocytes divert towards immunosuppressive and differentiation phenotypes

**Fig. 5:**
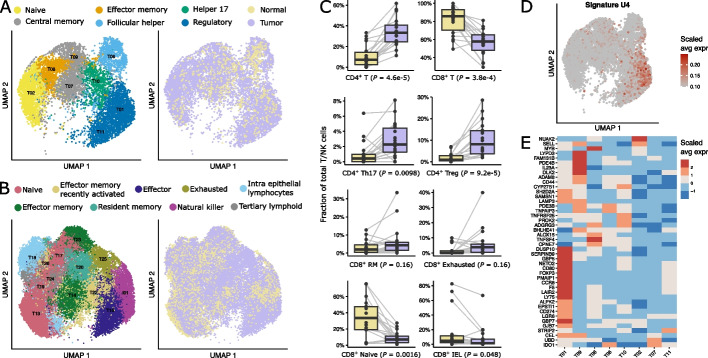
Immunosuppresive T cell dynamics in gastric cancer. **A,B** UMAP of 12,537 CD4^+^ T cells (**A**) and 28,772 CD8^+^ T cells (**B**) color coded for cluster annotation. **C** Pairwise analysis of T cell cluster fraction per patient in normal and tumor tissue, showing the most up- and downregulated T cells. Paired Wilcoxon rank-sum test, Holm-adjusted *p*-values per cluster shown. **D** Average expression of T cell implicated gene cluster U4 visualized on CD4^+^ T cell UMAP (Additional file 1: Fig. S3). **E** Heatmap of genes from gene cluster U4, color as scaled average expression

In contrast to CD8^+^ T cells, whose fraction stayed nearly unchanged between tumor and normal samples, we observed an over fivefold increase in CD4^+^ T cells in the gastric tumor samples. To understand which T cell subsets most differentially invaded the gastric TME, we reclustered the 12,905 CD4^+^ T cells into eight subclusters which we then assigned to six T helper cell subtypes including regulatory (*FOXP3*, *IL2RA*), helper 17 (*IL17A*), naïve (*CCR7*), central memory (CM) (*ANXA1*, *CCR7*), effector memory (EM) (*ANXA1*, *CCL5*), and follicular T helper cells (*CXCL13*) (Fig. 5a and S6a). In addition, the 31,705 CD8^+^ lymphocytes and 2,658 NK cells reclustered into thirdteen subclusters, 12 of which corresponded to eight different CD8 T cell subtypes and one corresponded to NK cells. The CD8^+^ T cells were designated as naïve (*IL7R*), effector (*GLNY*), effector memory (*ANXA1*, *GZMK*), effector memory expressing CD45RA (*LMNA*, *CREM*), resident memory (*ANXA1*, *GZMB*), exhausted (*HAVCR2*), intraepithelial (*CD160*) [46], and CD8^+^ T cells with a tertiary lymphoid signature (*CXCL13*) (Fig. 5b and Additional file 1: Fig. S6b). The expression profile associated with these T cell clusters closely resembled those of T cell subtypes reported in other cancer indications [47, 48]. Immunosuppressive Th17 and Treg cells were among the most increased CD4^+^ T cells while naive CD8^+^ T cells were reduced in tumors (Fig. 5c), likely reflecting the activation and expansion of T cells in the tumor. Besides gastric cancer, disproportionate CD4^+^ over CD8^+^ T cell ratios have also been observed in CRC [30, 49].

Our bulk and single-cell integration found one gene cluster U4 upregulated in gastric cancer that was preferentially expressed in T01-*ICOS* (Fig. 5d). Upon observing this cluster in more detail, we found many genes associated with activated regulatory T cell like *FOXP3* and *CCR8*, inhibition of apoptosis such as *PMAIP1,* and immune suppression such as *CD274*, *ALOX15*, and *SERPINB9* [50], emphasizing a strong activation of regulatory T cells and immune suppression in the gastric TME.

We identified 16,883 plasma cells and annotated them through gene signatures for heavy and light chain as well as kappa and lambda immunoglobulins, as IgAλ, IgAκ, IgGλ, IgGκ, IgMλ, IgMκ (Additional file 1: Fig. S7a). Comparing the ratio of plasma cell clusters between distal non-malignant gastric tissue and gastric tumor showed a high increase of IgG isotypes in gastric cancer whereas IgA isotypes decreased, suggesting a systemic change in the immune microenvironment (Additional file 1: Fig. S7b). The increase of IgG-positive plasma cells in gastric cancer is intriguing and has been documented in previous studies on other cancer indications [51].

### Cell communication network uncovers potential drivers of gastric cancer development

**Fig. 6:**
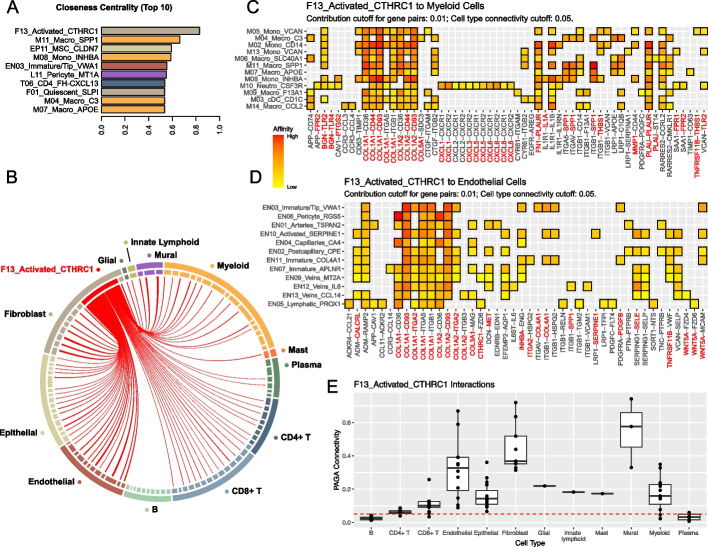
Cell communication inferred from single-cell transcriptome profiles in gastric cancer show a central role of F13-Activated-CTHRC1 fibroblasts. **A** Closeness centrality ranking of all cell subtypes in the inferred cluster-wise cell communication network, top 10 subtypes are shown. **B** Communication strength of F13-Activated-CTHRC1 fibroblasts with other cell subtypes, only significant communication (connectivity > 0.05) are drawn. **C** Significant ligand-receptor pairs in F13-Myeloid communication highlight tumor-enriched activation of integrin-Akt pathway in myeloid cells by F13-CTHRC1 cells. **D** Significant ligand-receptor pairs in F13-Endothelial communication show a tumor-restricted program for intercellular communication. The *X*-axis represent ligand and receptor pairs, with the first gene expressed on F13-Act-CTHRC1 cells and second gene expressed on the interacting cell types denoted in the *Y*-axis. Red: upregulated gene in tumor bulk RNA-seq. Blue: downregulated in tumor bulk (none present). **E** Communication strength of F13-CTHRC1 with other cell subtypes, red line denotes the cluster-wise connectivity cutoff of 0.05 (see “Methods”)

To investigate cellular interactions in the gastric cancer tumor microenvironment, we constructed a cell communication network based on ligand and matching receptor expression information from our single-cell atlas. The microenvironment was found to display a wide range of communications across different cell subtypes. Communication models derived from the cells of the TME and from normal gastric tissue thereby differed significantly in their structure, with different cell subtypes ranked most central in the interaction networks for TME and normal tissue, and F13-*CTHRC1* as a central network hub in the TME but not in normal tissue (Additional file 1: Fig. S8c-d). F13-*CTHRC1* thereby exhibited the highest centrality index in the TME network, with significant connections to many cell subtypes (Fig. 6a, S8a) including other fibroblasts, vasculature-associated endothelial cells, and immune cells (Fig. 6b, 6e). Many genes upregulated in gastric cancer participated in the F13-*CTHRC1* as well as in endothelial cell-specific interactions, while none of the downregulated genes were interacting, further pointing towards the existence of a tumor-specific cell communication program (Fig. 6d). Notably, F13-*CTHRC1* was predicted to communicate with the tumor-enriched EN10-*SERPINE1* and EN03-*VWA1* endothelial cells through *WNT5A-MCAM* and *LRP1-SERPINE1* interactions, both of which are highly expressed by these cell types and have been characterized as molecular switches for cell motility and angiogenesis [52, 53]. Moreover, by engaging endothelial cells and macrophages via integrin signaling, F13-*CTHRC1* might not only foster tumor remodeling through angiogenic stimulation but might also enhance the development of anti-inflammatory macrophages [54] (Fig. 6c). Finally, F13-*CTHRC1* harbors immunosuppressive interactions with T cells and DCs through *TSLP-IL7R* signaling, respectively (Additional file 1: Fig. S9c, S9d). TSLP is responsible for the expansion of regulatory T cells [55] and initiates T helper 2 responses in the tumor which are associated with worse survival prognosis [56]. Together, these findings indicate a potential role for F13-*CTHRC1* as driver of gastric cancer progression through angiogenesis-stimulating and immune suppressive interactions with endothelial cells and immune cells.

### Top ligand-receptor interactions between gastric tumor cells and the TME point to mechanisms of immune evasion

Although we applied EPCAM^+^ cell depletion before single-cell sequencing to enrich for cells of the TME, our dataset also contains 7210 epithelial cells. These cells clustered into fifteen groups (Additional file 1: Fig. S10a) which were annotated as gland mucus cells (GMCs) (MUC6 and TFF2), pit mucus cells (PMCs) (MUC5AC and TFF1), parietal cells (ATP4A and ATP4B), chief cells (PGA3 and PGA4), enterocytes (FABP1 and APOA1), and malignant cells (Additional file 1: Fig. S10a-b). The latter were identified via the copyKAT algorithm (Additional file 1: Fig. S10d) that quantifies the level of aneuploid chromosomal regions in cells. Malignant cells (designated EP11-MSC-CLDN7) were found only in the tumor samples (Additional file 1: Fig. S10a-b) and expressed the highest levels of EPCAM (Additional file 1: Fig. S10e) although more sporadic expression of this marker was also seen in the other epithelial cell clusters including enterocytes and GMCs, indicating that our EPCAM cell depletion has been incomplete. High EPCAM levels in EP11-CLDN7 correlated positively with the presence of cell cycle markers in this cluster further confirming the malignant nature of these cells. (Additional file 1: Fig. S10e).

Malignant cell cluster EP11-CLDN7 was connected to 33 cell types in the cell-interaction network (Fig. 6A). The top interacting cell types were thereby CD8^ +^ T cells as well as neutrophils and F13-CTHRC1 fibroblasts (Additional file 1: Fig. S10f). The interaction between EP11-CLDN7 and CD8^+^ T cells was dominated by the interactions of LGALS9—HAVCR2 and HVEM—CD160 both of which are well-known checkpoints that inhibit CD8^ +^ T cell functionality (Additional file 1: Fig. S10g), suggesting an immunosuppressive function of the gastric tumor cells. EP11-CLDN7 and F13-CTHRC1 cells are predicted to interact through FZD5–WNT5A, reinforcing the hypothesis of F13-CTHRC1 potentially facilitating tumor cell migration [57]. Lastly, neutrophils were connected to EP11-CLDN7 cells via chemokine-receptor pairs CXCL1/2/3/5/8–CXCR1/2 which could be an important axis for the attraction of immune suppressive neutrophils [58, 59].

### Cell type signatures predict patient outcome in an independent gastric cancer cohort

**Fig. 7:**
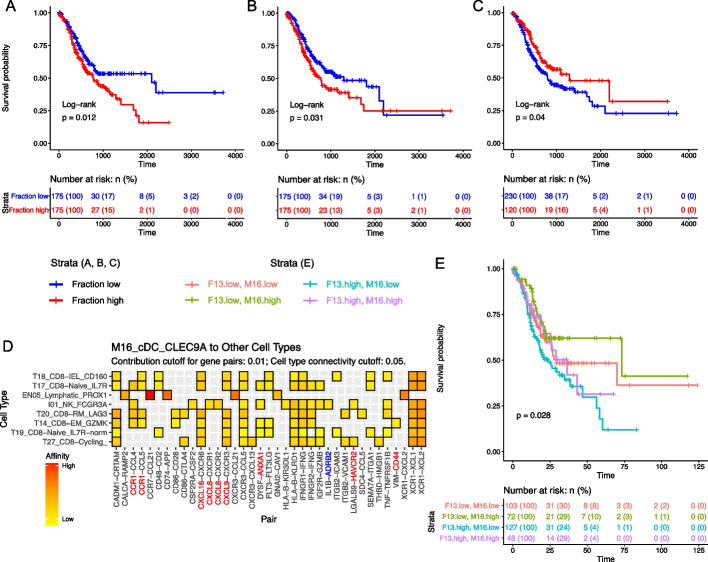
Deconvolution of bulk RNA-seq from an independent gastric cancer cohort reveal prognostic impact of cell subtypes. **A–C** Kaplan–Meier curve of TCGA Gastric Cancer patients with high or low F13-Activated-CTHRC1 (**A**), EN10-Activated-SERPINE1 (**B**), and M16-cDC-CLEC9A (**C**) scores. **D** Significant ligand-receptor pairs in M16-cDC-CLEC9A communication suggest CLEC9A + cDC1 supports antitumor immunity through XCL1-XCR1 signaling. The *X*-axis represents ligand and receptor pairs. The *Y*-axis represents cell subtypes interacting with M16-cDC-CLEC9A. **E** Kaplan–Meier curve of patients with high F13 score and low M16 score achieve best prognosis

Our precise mapping of diverse non-malignant cells in the gastric cancer microenvironment provided a comprehensive reference for interrogating the impact of different cell populations on patient prognosis from other types of data, especially the bulk transcriptome data from the TCGA gastric cancer cohort. Using transcriptomic information from our single-cell data as reference, we used CibersortX [57] to infer the fraction of each cell subtype in the TCGA samples and subsequently assessed the survival differences between patients with higher and lower fractions of each cell subtype. Notably, patients with higher fraction of tumor-associated cell subtypes F13-*CTHRC1* and EN10-*SERPINE1* had significantly reduced survival time (Fig. 7a, b). In addition, several other endothelial and epithelial subtypes also displayed significant negative impact on gastric cancer patient survival (Additional file 1: Fig. S8), pointing towards being potentially relevant in gastric cancer progression. In contrast, the fraction of cDC1 dendritic cells (M16-cDC-*CLEC9A*) had significant positive impact on patient survival (Fig. 7c). Although being a rather rare population in the tumor microenvironment, intratumoral cDC1 cells are considered critical for antitumor immunity as they attract, stimulate, and support tumor-infiltrating cytotoxic T cells through different molecular signals [60–62]. Consistent with this functional role [63], in our network analysis we found M16-*CLEC9A* to interact with a wide collection of CD8^+^ T cell and NK cell populations through a number of communication signals including most prominently the XCL1/XCL2-XCR1 receptor-ligand axis (Fig. 7d). Accordingly, combining these inversely correlated signatures resulted in an even more pronounced survival difference between F13-enriched/M16-depleted and F13-depleted/M16-enriched patients (Fig. 7e).

### Integration of gastric cancer immunotherapy bulk RNA-seq reveals cellular and molecular predispositions to response

**Fig. 8:**
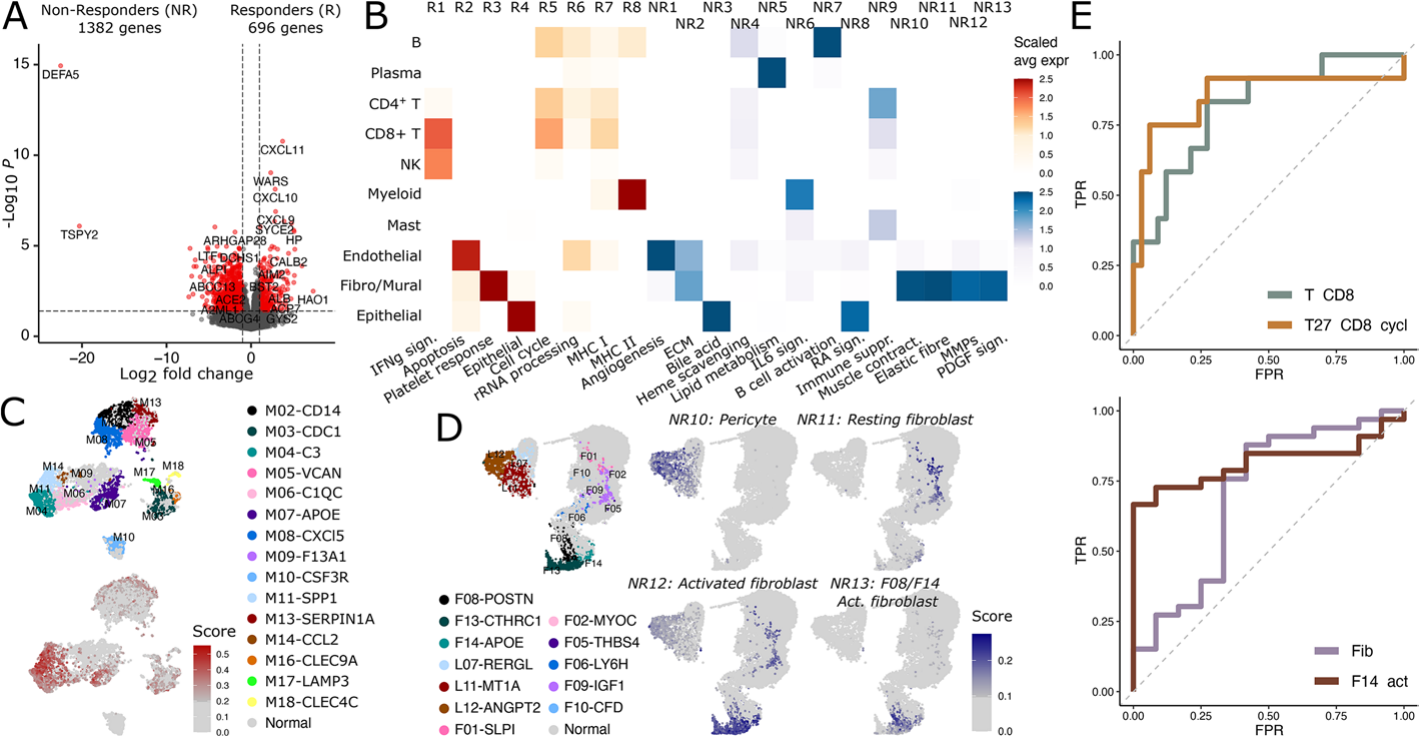
Cellular origin of response and non-response genes to cancer immunotherapy. **A** Differential expression analysis between responders (complete and partial responders, *n* = 12) and non-responders (stable and progressive disease, *n* = 33) performed on bulk RNA-seq from a total of 45 gastric cancer patients that underwent immunotherapy. Genes for downstream analysis (red) with adjusted *p*-value < 0.1 and log2 fold change > 0.25. **B** Up- and downregulated genes (**A**) were clustered based on their expression in minor cell types of the single-cell RNA-seq data (Additional file 1: Fig. S3). Here, a heatmap of the scaled average expression from the upregulated gene clusters is shown per major cell types of the single-cell RNA-seq on gastric cancer. **C,D** Annotation and UCell score of myeloid cells with gene cluster R8 (**C**) and fibroblasts with gene clusters NR10-13 (**D**) visualized on UMAP. **E** ROC curves of gene signature scores corresponding with specific cell subtypes on response data from cohort in **A**

Immunotherapy via blocking the PD1/PDL1 axis or via blocking CTLA4 signaling has become standard of care in several cancer indications and has yielded promising results in gastric cancer as well [64, 65]. However, many gastric cancer patients do not profit from ICI treatment [66]. To identify what cell types and TME signaling pathways might contribute to resistance to ICI treatment, we obtained bulk RNA-seq data from a recent gastric cancer study [6] that evaluated gene expression in tumor samples from 45 patients treated with anti-PD-1 (pembrolizumab) therapy. Differential gene expression analysis yielded 696 and 1382 genes significantly higher expressed in pretreated samples from the 12 responding and 33 non-responding patients, respectively (Fig. 8a).

Bi-clustering the set of 696 genes associated with response across our single-cell expression data yielded 8 gene groups, designated R1 to R8 (Fig. 8b, S12a). Cluster R1 was exclusively expressed in T cells and NK cells and was strongly enriched for genes associated with IFN-γ signaling [67]. Genes associated with peptide presentation via MHC I complexes (R7) [68] and MHC II complexes (R8) [69] were also found to be overexpressed in responders and specifically associated with lymphoid cells and myeloid cells, respectively (Additional file 1: Fig. S12b). Interestingly, cluster R8 contained prominent marker genes such as *C1QA* and *C1QB* and was most strongly expressed in M11-*SPP1* anti-inflammatory macrophages, suggesting a positive influence of these cells on immunotherapy response (Fig. 8c).

Bi-clustering of the 1382 non-responder genes in our single-cell data yielded 13 different clusters, designated NR1 to NR13, with high specificity to endothelial cells and fibroblast as well as epithelial and plasma cells (Fig. 8b, S12c). Gene set enrichment analysis identified as main associated pathways angiogenesis in endothelial cells, and IL6 signaling in myeloid cells (Additional file 1: Fig. S12d). Fibroblast have been linked to immunotherapy resistance in several cancer types [70, 71]. Correspondingly, here we found four different gene clusters, NR10, NR11, NR12, and NR13 highly specific for pericytes, resting fibroblasts, F13-*CTHRC1*, and activated fibroblast, respectively (Fig. 8d). Cluster NR12 was specifically enriched for pathways involving extracellular matrix degradation and expressed several metalloproteinases including *MMP9 *which has been shown to mediate anti-PD1 resistance in melanoma.

To understand the distribution of these gene clusters in gastric cancer, we calculated a signature score for all eight responder and 13 non-responder clusters for each of the 295 gastric cancer RNA-seq samples from the gastric cancer TCGA cohort. Hierarchical bi-clustering of the scores clearly separated response and non-response gene signatures and revealed differential expression of each signature across different patient groups (Additional file 1: Fig. S12e). Non-responder clusters NR3, NR5, and NR8 were thereby found to be clearly distinct from the other non-responder gene clusters likely due to their association with plasma and epithelial cells. Classifying the TCGA tumors based on recently identified molecular subtypes revealed that responder signatures were enriched in ICI sensitive EBV and MSI patients while non-response signatures were enriched in CIN and GS patients (Additional file 1: Fig. S12f).

To investigate if any of the 11 main cell types or 81 subtypes identified in the gastric TME could be used to predict response to immunotherapy, we analyzed the power of a binary classification model consisting of the top marker genes from each cell type (see “Methods”) to predict treatment outcome in the above gastric cancer immunotherapy cohort [6]. With an area under the ROC curve of 0.85, the presence of T27-cycling CD8^+^ T cells followed by the presence of pan CD8 T cells (AUROC of 0.81) was found to best predict treatment outcome in this cohort (Fig. 8e). Presence of F14-*ADAM2*8 activated fibroblasts followed by presence of pan-fibroblasts was best predictive for non-response with AUROCs of 0.82 and 0.7, respectively (Fig. 8e) and up to 100% specificity (Additional file 1: Fig. S12h-i and Additional file 7: Table S6).

## Discussion

In this study, we performed bulk and single-cell RNA sequencing on matching malignant and non-malignant samples from a total of 24 gastric cancer patients. By integrating bulk and single-cell RNA sequencing, we were able to link cancer-associated transcriptional programs to different cell populations. This approach also allowed to integrate many lowly expressed genes oftentimes not well detected in single-cell data such as *HOX9A* and *CXCL9* (Additional file 3: Table S2) which are important in cancer progression and T cell recruitment, respectively. One of the key molecular programs we found was associated with the strong immunosuppressive phenotype of T01-*ICOS* activated regulatory T cells and M17-*LAMP3* dendritic cells. Both T01-*ICOS* and M17-*LAMP3* cells were increased in the TME and formed strong immune suppressing interactions with CD8^+^ T cells via receptor-ligand interactions such as *CTLA4/CD80* and *CD274/PDCD1*. Furthermore, mapping genes associated with response to anti-PD1-treatment to our single-cell atlas revealed cell types that separate ICI responders from non-responders. Whereas immune activation and antigen presentation connected to CD8^+^ T cells and dendritic cells were overexpressed in patients responding to immunotherapy, non-responding tumors featured upregulated angiogenic, matrix remodeling, and pro-tumorigenic signaling pathways connected to activated fibroblasts and endothelial cells. In general, we did not observe significant differences in the TME based on ethnicity or tumor mutational burden. However, we found the marker gene signature from F14-*ADAM2*8 fibroblasts to be differentially expressed in genomically stable patients (Additional file 1: Fig. S12j-k), who in general do not respond to immunotherapy treatment.

Key molecular features and cell subtypes identified in this study resembled previous findings from both gastric as well as other cancer indications. For instance, in line with our results, a recently published atlas of gastric cancer TME also found RSPO3 signaling in fibroblasts and Notch signaling in endothelial cells (Additional file 1: Fig. S13). Another cell compendium of cross-tissue fibroblast [18], described steady-state fibroblast, positive for *PI16* and *COL15A1*, which were strikingly similar to resting fibroblast in our gastric cancer data. Steady-state fibroblasts in cancer were described to progress towards activated CAFs expressing for instance *CTHRC1*, and *COL11A1*, similar to the differentiation trajectory we found for F13-*CTHRC1* activated fibroblasts in gastric cancer. Presence of activated fibroblasts has also been correlated with lack of response to immunotherapy in pancreatic and breast cancer [70, 72]. Besides activated fibroblasts, we also found two clusters of activated endothelial cells in gastric cancer. Activated endothelial subtypes have been documented in prostate cancer and were shown to express CAF markers [73]. These activated endothelial cell subtypes were enriched with angiogenic and matrix remodeling pathways and progressively increased in number with cancer stage, highlighting their role in invasion and aggressive cancer advancement. Another common theme not only observed in gastric cancer but in the TME from various indications including breast, liver, colorectal, and lung cancer is the appearance of a cluster of activated and immune suppressive regulatory T cells expressing activation markers such as 4-1BB and CCR8 [30, 35, 47, 74]. These CCR8-positive activated Tregs possessed strong immunosuppressive functionality through production of immune suppressive metabolites like extracellular AMP, as well as blocking CD80/CD28 signaling via high expression of *ICOS* and *CTLA-4.* They also have been associated with stromal cell activation and communication with CAFs through CD73, DPP4, and B7H3 [75]. Activated forms of dendritic cells and macrophages in the TME have been reported as a cluster of LAMP3-positive dendritic cells in hepatocellular carcinoma and colorectal cancer [34, 35] and as SPP1-positive macrophages in colorectal cancer [30], respectively. LAMP3-positive dendritic cells mediated immunosuppression through the expression of immune checkpoints *CD274* and *IDO1* in gastric cancer, while SPP1-positive macrophages expressed both proinflammatory as well as anti-inflammatory signatures making their role in gastric tumor progression unclear and in need of further investigation.

## Conclusions

Here, we presented an encyclopedic view of tissue-resident and infiltrating cells in human gastric tumors and matched non-malignant stomach tissue at single-cell resolution. We analyzed a total of 96,623 cells derived from samples of 24 gastric cancer patients and identified 81 different cell subtypes associated with the gastric tumor microenvironment. This comprehensive single-cell atlas allowed us to identify not only changes in cell type frequencies between tumor and normal gastric tissue but also changes in the transcriptional programs associated with malignant transformation. We found the gastric TME to be marked by a significant remodeling of its stromal component with EN10-*SERPINE1* endothelial cells and F13-*CTHRC1* activated fibroblasts representing tumor-specific cell populations. Our cell communication network revealed crosstalk between EN10-*SERPINE1* and F13-*CTHRC1* and proposes interaction axes that could play a role in angiogenesis, migration, and facilitating epithelial to mesenchymal transition of the gastric tumor cells [22, 23, 76, 77]. Using our single-cell atlas to deconvolute the bulk RNA-seq data from gastric cancer cohort from  TCGA, we found an association of certain dendritic and stromal cell subtypes to patient survival. While high levels of M16-CLEC9A dendritic cells were associated to a prolonged survival, high numbers of F13-*CTHRC1* activated fibroblasts and EN10-*SERPINE1* endothelial cells were correlated with poor survival for gastric cancer patients. In addition, our bulk/single-cell integration applied to a PD1-treated gastric cancer cohort identified gene programs expressed in CD8^+^ T cells and activated fibroblasts of the TME that contrastingly influenced the outcome of immunotherapy response. Together, these findings suggest novel opportunities for predictive biomarkers. For example, we showed that a gene signature of cycling CD8^+^ T cells was highly predictive for response to immunotherapy especially in GS and CIN patients. Furthermore, non-responder gene programs were associated with stromal, plasma, and EPCAM-negative PMCs as well as EPCAM-positive malignant epithelial cells and highlight potential targets for patient selection or therapeutic intervention. The presence of tumor-specific activated stromal cells in our data suggest the applicability of cell type depletion strategies that are currently under development such as LRRC15 [78] and CCR8 [79] antibody drug conjugates in gastric cancer. The data also suggests novel therapeutic directions such as blocking IL-6 signaling from myeloid cells and plasminogen activation in activated endothelial cells for treating gastric cancer.

## Methods

### Gastric cancer sample collection

Twenty-four gastric cancer patients were enrolled in this study. None of the patients was treated with chemotherapy, radiation, or any other antitumor medicines prior to tumor resection (Additional file 2: Table S1). This study was approved by Peking University IRB. All patients enrolled in this study provided written informed consent for sample collection and data analyses. The experimental methods comply with the Declaration of Helsinki. Fresh tumor and adjacent normal tissue samples (at least 2 cm from matched tumor tissues) were surgically resected from the above-described patients and shipped in RPMI-1640 medium (Gibco) at 4 °C. The available clinical characteristics and sequencing chemistry information are summarized in Additional file 2: Table S1.

### Bulk WES and RNA-seq analysis

WES and RNA-seq are performed on bulk samples separated from the collected tissue. WES was performed using the Agilent V6 Human Exome Capture Chip, with the sequences aligned and annotated using the GATK 4.0 [80] pipeline and GRCh38 reference genome. Copy number variation of tumor sample is subsequently computed using Control-FREEC (v5.7) [81] against the paired normal sample, using parameters breakPointType = 4, breakPointThreshold = 1.2, noisyData = TRUE, readCountThreshold = 50. RNA-seq was performed using the RNeasy Mini Kit (QIAGEN), with the sequences quantified using Kallisto (v0.45.0) [82] against the GRCh38 reference genome (Ensembl 93 annotation). Downstream tumor-normal differential expression analysis was performed using R package DESeq2 [83], using the parameters adjusted *p*-value < 1 × 10^−5^, log_2_FC > 2.

### Single-cell dissociation, sorting, library preparation, and sequencing

Tumors and adjacent non-cancer tissues were cut into approximately 1–2-mm^3^ pieces in the RPMI-1640 medium (Gibco) with 10% fetal bovine serum (FBS, GIBCO), and enzymatically digested with gentleMACS (Miltenyi) for 60 min on a rotor at 37 °C, according to the manufacturer’s instruction. The dissociated cells were subsequently passed through a 100-µm SmartStrainer and centrifuged at 400* g* for 5 min. After the supernatant was removed, the pelleted cells were suspended in red blood cell lysis buffer (TIANDZ) and incubated on ice for 1–2 min to lyse red blood cells. After washing twice with 1 × PBS (Gibco), the cell pellets were re-suspended in sorting buffer (PBS supplemented with 1% fetal bovine serum (FBS, Gibco)).

Single-cell suspensions were stained with antibodies against CD326 (EPCAM, BioLegend, Cat #324,207) and 7-AAD (eBioscience, Cat# 00–6993-50) for FACS sorting, performed on a BD Aria SORP instrument. Expression levels of EPCAM and permeability of 7-AAD were gated by their negative controls of unstained cells and positive controls of beads stained by each antibody. Based on FACS analysis, single cells were sorted into 1.5 ml tubes (Eppendorf) and counted manually under the microscope. The concentration of single-cell suspensions was adjusted to 500–1200 cells/µl. Cells were loaded to the 10 × Chromium Microfluidic Chips for single-cell RNA and TCR library preparation. All the subsequent steps were performed following the standard manufacturer’s protocols. Purified libraries were analyzed by an Illumina Hiseq-4000 sequencer with 150-bp paired-end reads.

### Single-cell sequencing data processing

The 10X droplet-based single-cell RNA sequencing data were processed using CellRanger toolkit (version 3.0.0) provided by 10X Genomics. Gene expression levels are quantified using GRCh38 reference genome (Ensembl 93 annotation). For each cell identified by CellRanger, we calculated the total number of detected genes, total number of UMI counts, and proportion of mitochondrial reads. A set of quality thresholds was applied to filter out low-quality cells, including detection of 200–7500 genes, 500–75,000 UMI counts, and less than 10% mitochondrial reads, resulting in a total cell number of 117,506 post-filter cells that were used for clustering analysis.

### Normalization and batch effect correction

Cells passing quality filter were normalized with SCTransform [84] using the default parameters. Independent component analysis (ICA) was applied on the normalized gene-cell matrix to identify potential batch effects. Out of 128 independent components, an independent component (IC_15) was found to have a highly sample-specific distribution (Additional file 1: Fig. S1b). We further inspected the top weighted genes in this independent component and found this IC populated by a heat-shock protein-related program (Additional file 1: Fig. S1c), potentially derived from enzymatic stimulation during tissue dissociation [85]. The gene expression program driven by IC_15 was then subtracted from the normalized gene-cell matrix to remove this dissociation-derived batch effect.

### Stepwise integration and unsupervised clustering analysis

To achieve stable and un-biased identification of cell populations, we used a stepwise approach implemented with SCANPY [86] for cell clustering. First, using top 1000 genes with the highest variance selected from the normalized cell-gene matrix, principal component analysis (PCA) was applied on the global population to reduce dimensionality to 100 principal components. Next, BBKNN [87] was applied to construct a balanced *k*-nearest neighbor graph across the 10X 3′ and 5′ chemistries (Additional file 1: Fig. S1e). Then, the Leiden clustering algorithm was applied on the balanced KNN and identified 25 clusters. One cluster was identified as low-quality and excluded for downstream analyses according to its high mitochondrial content, resulting in a total number of 96,623 cells used for downstream analyses (Additional file 1: Fig. S1d). To prevent over-clustering, the rest 24 clusters were merged into 12 major populations (B, plasma, mast, myeloid, epithelial, endothelial, fibroblast, CD4 T, CD8 T/NK, Cell Cycle CD4 T, cell cycle CD8 T, cell cycle B) based on stable expression of their canonical markers.

Next, the normalized cell-gene matrix of each major population was extracted for identification of subpopulations. The top 600 variable genes were used for PCA, and the first 25–30 principal components were used for BBKNN. Leiden clustering algorithm was applied to identify distinct subpopulations from the major population. When clusters are determined, the Wilcoxon rank-sum test was used to identify differentially expressed genes (DEGs) among clusters. Genes are considered differentially expressed if the Benjamini-Hochberg-adjusted *p*-value < 0.05, and the fraction of cells expressing the gene is over 30% (Additional file 3: Table S2). Some clusters with low median of expressed genes (around 200) and that did not yield any unique DEGs were annotated as low-quality and removed from subsequent analyses resulting in a total of 81 minor clusters, these clusters were as follows: F04, F15, B03, B08, T03-05, T16, T21 EN08, EN09, EN11, EN13, EP12, and M15. Each subpopulation (cluster) was then annotated according to their gene expression profile (Additional file 1: Fig. S1f). Due to the small population size and homogeneous gene expression profiles of mast, cell cycle B, cell cycle CD4 T, and cell cycle CD8 T cells, this second round of cell clustering was not applied on them. The states of fibroblast subclusters were further analyzed by cNMF [19] using the recommended *k* selection criteria and the default parameters. After cNMF decomposition, *k* = 11 was chosen, and the weights (usages) of 11 expression modules in every cell were then summarized for subclusters using arithmetic mean. For plotting (Additional file 1: Fig. S3B), the average module weight was *z*-score scaled for each module.

Dimensionality reduction analysis applied to the expression data revealed that cells clustered primarily based on their tissue origins and subtypes but not based on patient origin. To further evaluate cluster compositions, we counted the number of patient samples contributing to each of our 81 minor cluster and found that all clusters contained samples from at least five or more patients (Additional file 1: Fig. S1g).

### Bi-clustering for integration of bulk and scRNA-seq data

Differentially expressed genes were first identified from bulk RNA-seq data between the matched non-malignant and malignant bulk samples (Additional file 5: Table S4) and responders vs non-responders of an anti-PD-1-treated gastric cancer cohort (Additional file 5: Table S5). The significantly up- and downregulated genes were then bi-clustered according to their expression across the single-cell landscape. Gene mapping from differentially expressed genes were mapped onto the single-cell expression data using the function plotMarkerHeat from the genesorteR R package [88], setting averageCells to 10^6^ and clusterGenes to TRUE.

To assess the robustness of the results obtained by the bi-clustering approach against changes in cell frequencies in the single-cell data, we applied the algorithm to 100 downsampled versions of our dataset where each time 90% of the fibroblast cells had been randomly selected and removed. Results obtained from the original and downsampled data were found to be highly similar with almost all clusters preserving at least 75% of the genes that were originally assigned to them (Additional file 1: Fig. S4f-h).

### Cell communication analysis

We used the method described by Ren and colleagues [89] to estimate the cell–cell affinity. Briefly, the cell–cell affinity contributed by a ligand pair *L*_*i*_ and *R*_*i*_ expressed by cell C1 and cell C2 is defined by the following formula:

$${\mathrm{Affinity}}_{\mathrm{C}1,\mathrm{C}2,Li,Ri}={E}_{\mathrm{C}1,Li} \times {E}_{\mathrm{C}2,Ri}$$

And the total affinity between cell C1 and cell C2, contributed by all ligand-receptor pairs:

$${A}_{C1,C2}={\sum_i}\left({E}_{\mathrm{C}1,Li}\times {E}_{\mathrm{C}2,Ri}\right)+{\sum_i}\left({E}_{\mathrm{C}1,Ri}\times {E}_{\mathrm{C}2,Li}\right)$$

where *L*_*i*_ and *R*_*i*_ denote the *i*th ligand and receptor in all the ligand-receptor pairs used for computation, *E*_C1*,Li*_ denotes the normalized expression of *L*_*i*_ in cell C1, and *E*_C1,*Ri*_ denotes the normalized expression of *R*_*i*_ in cell C1. The SCTransform-normalized gene expression values were used in the calculation of cell–cell affinities. After generation of the cell–cell affinity matrix, a modified *k*-nearest neighbor network was constructed based on strongest cell–cell affinities. Then, partition-based graph abstraction (PAGA) [90] was applied on the cluster groupings assigned in the stepwise clustering process to quantify cluster-wise communication strength. Inter-cluster connections with connectivity > 0.05 were considered significant.

For identification of important ligand and receptor pairs in the cluster-wise communication, the average affinity and contribution of a ligand-receptor pair *L*_*1*_*R*_*1*_ in the total interaction between two clusters *M* and *N* was calculated by the following formulae:

$${\mathrm{Affinity}}_{L\mathit1\mathit,R\mathit1\mathit,M\mathit,N}=\;{\sum_{m,n}}\left({\mathrm{Affinity}}_{Cm\mathit,Cn\mathit,L\mathit1\mathit,R\mathit1}\right)\div m\div n$$

$${\mathrm{Contribution}}_{L\mathit1\mathit,R\mathit1\mathit,M\mathit,N}=\;{\mathrm{Affinity}}_{L\mathit1\mathit,R\mathit1\mathit,M\mathit,N}\;\div\;{\sum_i}\;\left({\mathrm{Affinity}}_{Li\mathit,Ri\mathit,M\mathit,N}\right)$$

where *Cm *$\in$* M*, *Cm *$\in$* N*. Ligand-receptor pairs with a contribution > 0.01 are plotted.

### Deconvolution of RNA-seq data

Fraction of cell subtypes in TCGA-STAD was inferred using CIBERSORTx. FPKM and meta data of TCGA-STAD RNA-seq samples were downloaded from UCSC Xena Browser (https://xenabrowser.net/). Single-cell reference data were prepared by sampling 100 cells per cluster from the normalized gene expression matrix, then processed with the CIBERSORTx Create Signature Matrix function using default parameters. Fractions of the cell subtypes in each TCGA-STAD sample were then calculated using the CIBERSORTx Impute Cell Fractions function in the relative mode using default parameters.

To assess the accuracy of deconvoluting 81 minor cell types with CIBERSORTx, we created a pseudobulk dataset from our scRNA-seq and calculated cell fractions based on the same procedure as described above. We first checked how well the CIBERSORTx predictions for our 10 major cell types correlate with the actual single-cell fractions in our study. All cell types showed highly significant Pearson correlations (Additional file 1: Fig. S14a) between predictions and actual cell type frequencies. We then looked into the prediction accuracy of CIBERSORTx for the 81 cell subtypes. As shown in Additional file 1: Fig. S14b, results were in highly significant agreement with the preselected single-cell abundances for the vast majority of the cell subtypes. Over 80% of the cell subtype frequencies predicted by CIBERSORTx had a Pearson correlation higher than 0.5 with the actual data (Additional file 1: Fig. S14b). These include the cell subtypes F13, EN10, and M16 which we highlight in the paper. For these cell types, Pearson correlation coefficients of 0.86, 0.74, and 0.59 were obtained, respectively (Additional file 1: Fig. S14b). Only 18 cell types had correlation coefficients below 0.5. We noticed that these cell types either had very low abundance close to the detection limit (Additional file 1: Fig. S14c) or possessed only few specific marker genes as is the case for some plasma cell types. Overall, we were astonished by the accuracy of the results and believe they demonstrate that marker gene-based deconvolution via CIBERSORTx can successfully be used to deconvolute the presence of most cell types in bulk RNA-seq data.

### Survival analysis

For each cell subtype, TCGA-STAD patients were divided into two groups (fraction-high and fraction-low) based on median of the CIBERSORTx inferred cell fractions in bulk RNA-seq samples. The Kaplan-Meyer curve and log-rank test (Mantel-Cox test) *p*-values of overall survival were used to quantify the difference of fraction-high and fraction-low groups in survival time. Cox-PH model was applied to compute the hazard ratio of each grouping based on inferred cell fractions. For combined survival analysis of two cell subtypes, patients were divided into four groups (A-high + B-high, A-high + B-low, A-low + B-high, A-low + B-low, where A and B are two cell subtypes) based on median of the inferred cell fractions. This analysis was performed using the R package survival (version 3.2) and survminer (version 0.4.8). The survival analysis of single genes or signatures (Figs. 2e, and 3d) on TCGA-STAD cohort was performed using Gepia2 [91] with default parameters.

### Tumor—normal cell cluster abundance comparison

Before conducting tumor to normal comparisons, quality control was performed and patient samples with less than 200 cells (P180305N, P190125N, and P180606N) were removed from the dataset (Additional file 1: Fig. S1a). In addition, for ratio calculations on myeloid cells, fibroblast/mural cells, endothelial cells, and T cells, samples with less than 50 cells of the corresponding cell type were excluded from the analysis of the corresponding cell type. To normalize for differences in the total cell count per sample, we next calculated the percentage of every cell type per sample. To identify cell types whose abundance changes with malignant transformation, we then performed a Wilcoxon rank-sum test (figures S2b, S2h, S5, and S10c) on samples from normal and tumor tissue. To visualize cell type abundances, we finally calculated a fold change between tumor and normal based on the average of the cell type percentages across all included patient samples.

### Analysis of gene set scores

Gene signature scores were calculated with the AddModuleScoreUCell function from the UCell [92] package using default parameters.

### ROC analysis

Classification of the top marker genes from all 81 subtypes on a gastric cancer bulk RNA-seq cohort with response information was performed with the R package ROCit. Top marker genes were defined by taking the three highest ranked genes by highest average fold change and lowest Wilcoxon rank-sum test with Holm correction of the minor subtype compared to the other subtypes.

### Gene set enrichment

Gene set enrichment was performed with the msigdbr package on the C2 reactome and C5 gene ontology biological processes.

## Supplementary Information

Additional file 1: Figure S1. Study design and processing, related to Fig 1. Figure S2. Stromal cells in gastric cancer and other cancer indications, related to figure 2. Figure S3. cNMF analysis and expression of ECM-related genes of fibroblast subtypes. Figure S4. Bulk and single-cell RNA-seq integration. Figure S5. Myeloid cluster abundance in malignant and non-malignant stomach. Figure S6. T cell clusters and marker genes, related to figure 5. Figure S7. Plasma cells in gastric cancer. Figure S8. Different communication models of cell clusters in normal stomach and gastric cancer. Figure S9. F13-CTHRC1 as a central hub for interaction in the gastric tumor microenvironment. Figure S10. Epithelial cells in malignant and non-malignant stomach samples. Figure S11. Volcano plot of all hazard ratios for every cell subtype deconvoluted from STAD TCGA cohort (methods). Cox regression with Wald test, significant subtypes in red (*p* <= 0.05). Figure S12. Cellular origin of response and non-response genes to cancer immunotherapy, related to Fig. 8. Figure S13. Expression of Wnt and Notch signaling genes in major cell types. Figure S14. CIBERSORTx performance on major and minor cell subtypes.

Additional file 2: Table S1. Patient characteristics.

Additional file 3: Table S2. Differential expression of 96 cell subtypes.

Additional file 4: Table S3. Differential expression of normal and tumor endothelial cells.

Additional file 5: Table S4. Differential expression of malignant and non-malignant bulk RNA-seq samples.

Additional file 6: Table S5. Differential expression of responders and non-responders in an anti-PD1 treated gastric cancer cohort.

Additional file 7: Table S6. Gene signature ROC classifier scores.

Additional file 8. Review history.

## Data Availability

Processed single-cell RNA-seq data is deposited in the GEO repository under accession (GSE206785, http://ncbi.nlm.nih.gov/geo/query/acc.cgi?acc=GSE206785) [93]. The raw FASTQ files of this study is deposited in GSA under accession (HRA002336, https://ngdc.cncb.ac.cn/gsa-human/browse/HRA002336) [94] and will be provided for scientific research upon request complying with the law due to Human Genetic Resources Regulation of China (https://ngdc.cncb.ac.cn/gsa-human/policy/policy.jsp). To access the raw data, researchers can apply to the Data Access Committee HDAC001238 through the GSA portal, providing their organization and intended research scope for the DAC to verify compliance with the Regulation. We estimate a response to be made in 2 weeks. Bulk RNA-seq datasets of TCGA-STAD can be obtained from XENA data portal (http://xena.ucsc.edu/) [95]. Bulk RNA-seq data of anti-PD1-treated gastric cancer patients can be found on the European Nucleotide Archive repository (PRJEB25780, http://www.ncbi.nlm.nih.gov/bioproject/?term=PRJEB25780) [96]. Code used for all processing and analysis is available under BSD 3-Clause License at Github (https://github.com/chriscainx/gastric-cancer) [97]. Additional and processed data is available at Zenodo [98].
