# Supplementary material for: Parallel single-cell and bulk transcriptome analyses reveal key features of the gastric tumor microenvironment
Source: Genome Biol. 2022 Dec 22;23:265. doi: 10.1186/s13059-022-02828-2 (PMC9773611; doi:10.1186/s13059-022-02828-2)
Supplement: Supplementary file 1 — Additional file 1: Figure S1. Study design and processing, related to Fig 1. Figure S2. Stromal cells in gastric cancer and other cancer indications, related to figure 2. Figure S3. cNMF analysis and expression of ECM-related genes of fibroblast subtypes. Figure S4. Bulk and single-cell RNA-seq integration. Figure S5. Myeloid cluster abundance in malignant and non-malignant stomach. Figure S6. T cell clusters and marker genes, related to figure 5. Figure S7. Plasma cells in gastric cancer. Figure S8. Different communication models of cell clusters in normal stomach and gastric cancer. Figure S9. F13-CTHRC1 as a central hub for interaction in the gastric tumor microenvironment. Figure S10. Epithelial cells in malignant and non-malignant stomach samples. Figure S11. Volcano plot of all hazard ratios for every cell subtype deconvoluted from STAD TCGA cohort (methods). Cox regression with Wald test, significant subtypes in red (p <= 0.05). Figure S12. Cellular origin of response and non-response genes to cancer immunotherapy, related to Fig. 8. Figure S13. Expression of Wnt and Notch signaling genes in major cell types. Figure S14. CIBERSORTx performance on major and minor cell subtypes. [file 13059_2022_2828_MOESM1_ESM.pdf]

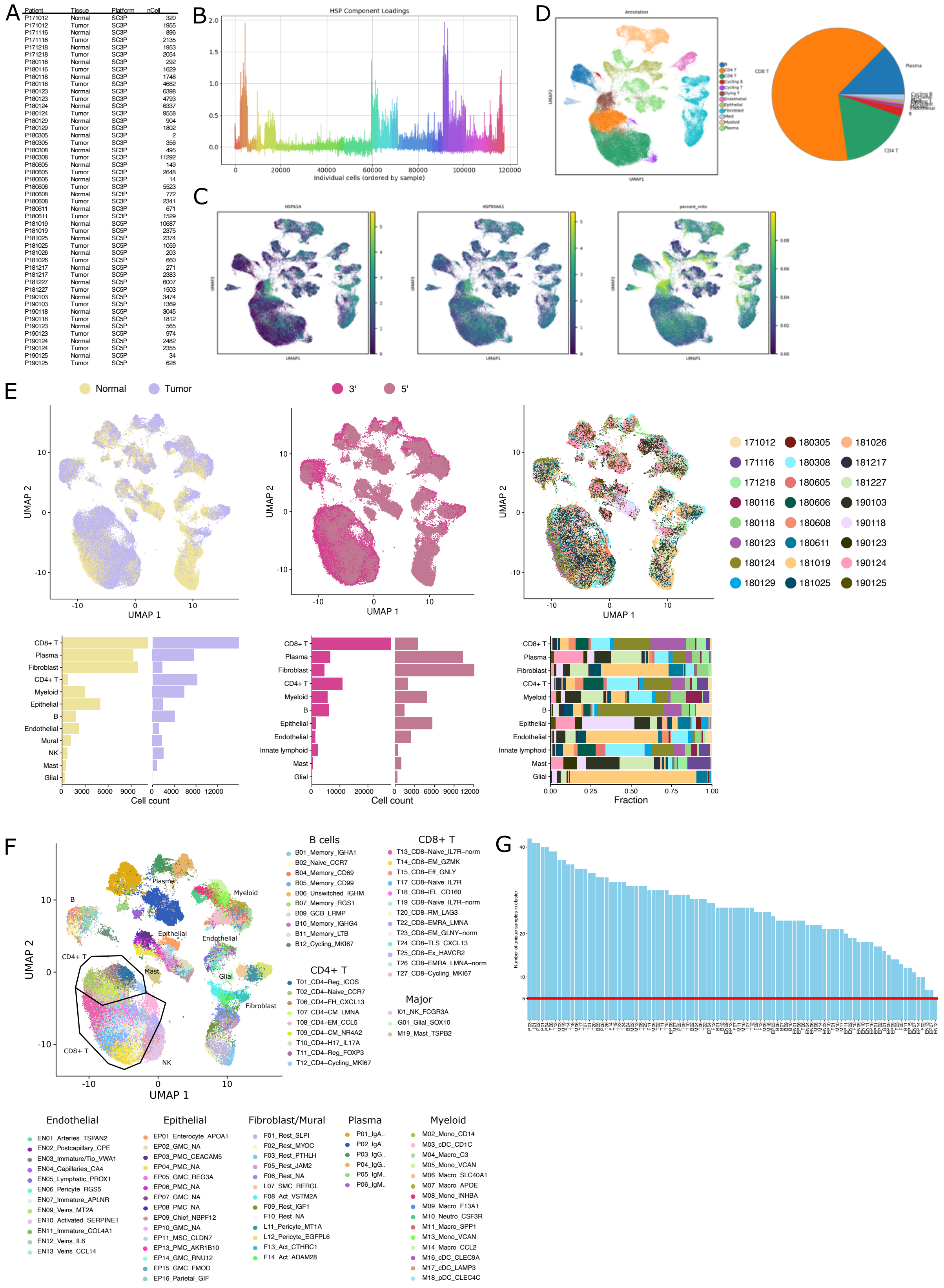

A

Patient	Tissue	Platform	nCell
P171012	Normal	SC3P	320
P171012	Tumor	SC3P	1955
P171116	Normal	SC3P	896
P171116	Tumor	SC3P	2135
P171218	Normal	SC3P	1953
P171218	Tumor	SC3P	2054
P180116	Normal	SC3P	292
P180116	Tumor	SC3P	1629
P180118	Normal	SC3P	1748
P180118	Tumor	SC3P	4682
P180123	Normal	SC3P	6398
P180123	Tumor	SC3P	4793
P180124	Normal	SC3P	6337
P180124	Tumor	SC3P	9558
P180129	Normal	SC3P	904
P180129	Tumor	SC3P	1802
P180305	Normal	SC3P	2
P180305	Tumor	SC3P	356
P180308	Normal	SC3P	495
P180308	Tumor	SC3P	11292
P180605	Normal	SC3P	149
P180605	Tumor	SC3P	2648
P180606	Normal	SC3P	14
P180606	Tumor	SC3P	5523
P180608	Normal	SC3P	772
P180608	Tumor	SC3P	2341
P180611	Normal	SC3P	671
P180611	Tumor	SC3P	1529
P181019	Normal	SC5P	10687
P181019	Tumor	SC5P	2375
P181025	Normal	SC5P	2374
P181025	Tumor	SC5P	1059
P181026	Normal	SC5P	203
P181026	Tumor	SC5P	660
P181217	Normal	SC5P	271
P181217	Tumor	SC5P	2383
P181227	Normal	SC5P	6007
P181227	Tumor	SC5P	1503
P190103	Normal	SC5P	3474
P190103	Tumor	SC5P	1369
P190118	Normal	SC5P	3045
P190118	Tumor	SC5P	1812
P190123	Normal	SC5P	565
P190123	Tumor	SC5P	974
P190124	Normal	SC5P	2482
P190124	Tumor	SC5P	2355
P190125	Normal	SC5P	34
P190125	Tumor	SC5P	626

Figure S1: Study design and processing, related to Fig 1.

A: Table with patient information and number of cells processed, patients with less than 50 cells were removed from further analysis. B: Cell loadings of heatshock protein (HSP) component color coded per patient. C: UMAP of 117,000 cells, single cells colored by HSP expression or percentage of mitochondrial reads. D: UMAP plot with annotation of major cell types (left) and predicted annotation of the dying T cell cluster (right). E: UMAP (top) and bar plot (bottom) of 96,623 cells, color coded from left to right for tissue of origin, processing platform and corresponding patient. F: 96 subtypes visualized on UMAP. G: Number of samples per minor cluster in full dataset, the bar indicates the average number of biological replicates. The red line indicates the cluster with the lowest number of samples (5 replicates).

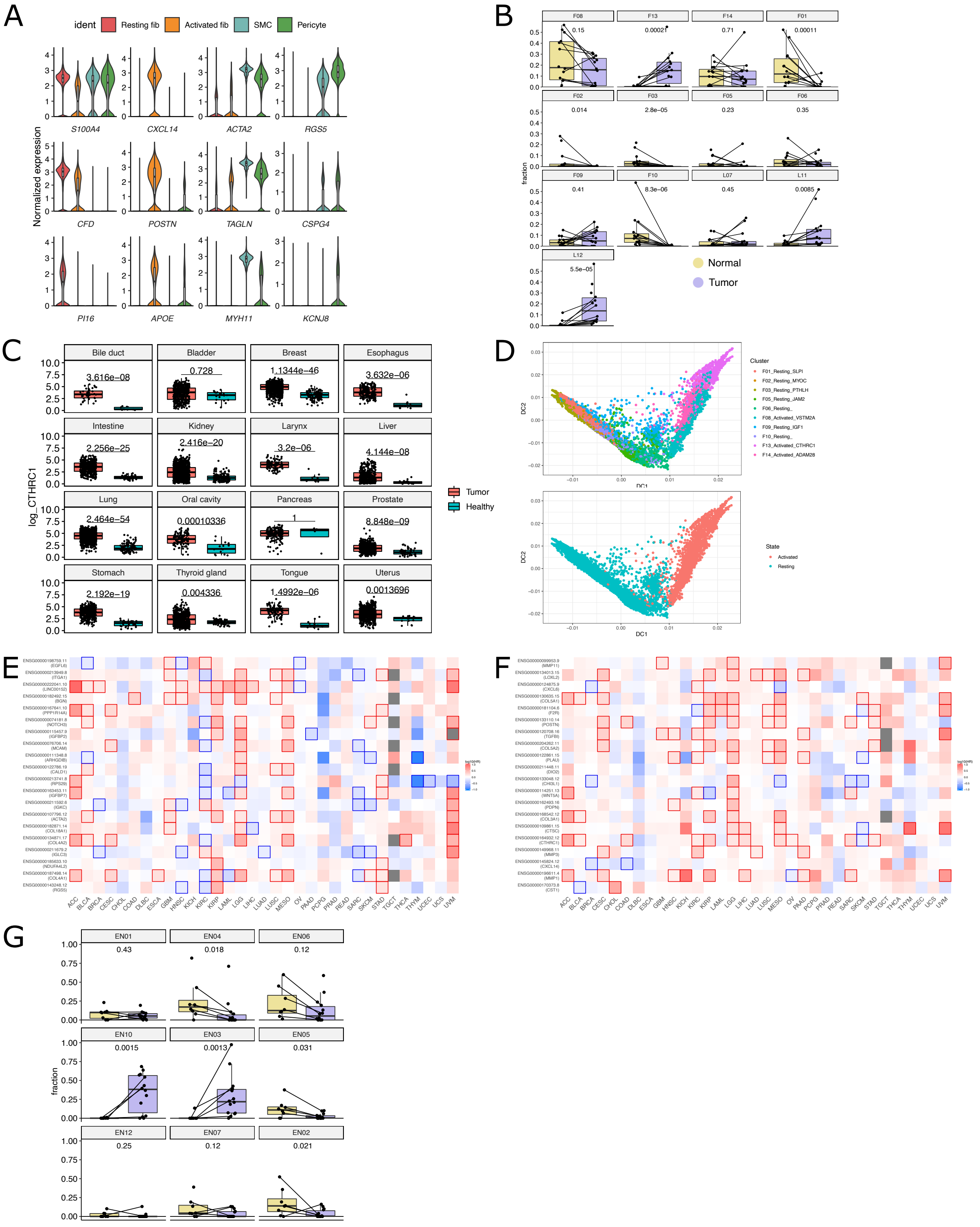

Figure S2: Stromal cells in gastric cancer and other cancer indications, related to figure 2.

A: Normalized expression of specific fibroblast markers corresponding to resting fibroblast, activated fibroblast, SMCs and pericytes annotation from Fig. 2A. B: Pairwise analysis of fibroblast and mural cell cluster fraction per patient in normal and tumor tissue, showing the most up and downregulated

cells. Paired Wilcoxon rank sum test, holm-adjusted *p* values per cluster shown, samples from similar patients connected. C: Expression of *CTHRC1* in bulk RNA-seq data of cancer indications and healthy tissue. Expression of *CTHRC1* shown as log TPM. P-values are calculated from a Wilcoxon-test with Bonferroni correction. D: Diffusion map analysis for resting and activated fibroblasts color coded for cluster annotation (top), with resting on the left and F13-*CTHRC1* fibroblast on the right, and cell state (bottom). E-F: Hazard ratio of patients with different cancer indications in TCGA, for top F12-*EGFL6* top 20 genes (E) and F13-*CTHRC1* top 20 marker genes (F). G: Pairwise analysis of endothelial cell cluster fraction per patient in normal and tumor tissue, showing the most up and downregulated cells. Paired Wilcoxon rank sum test, holm-adjusted *p* values per cluster shown, samples from similar patients connected.

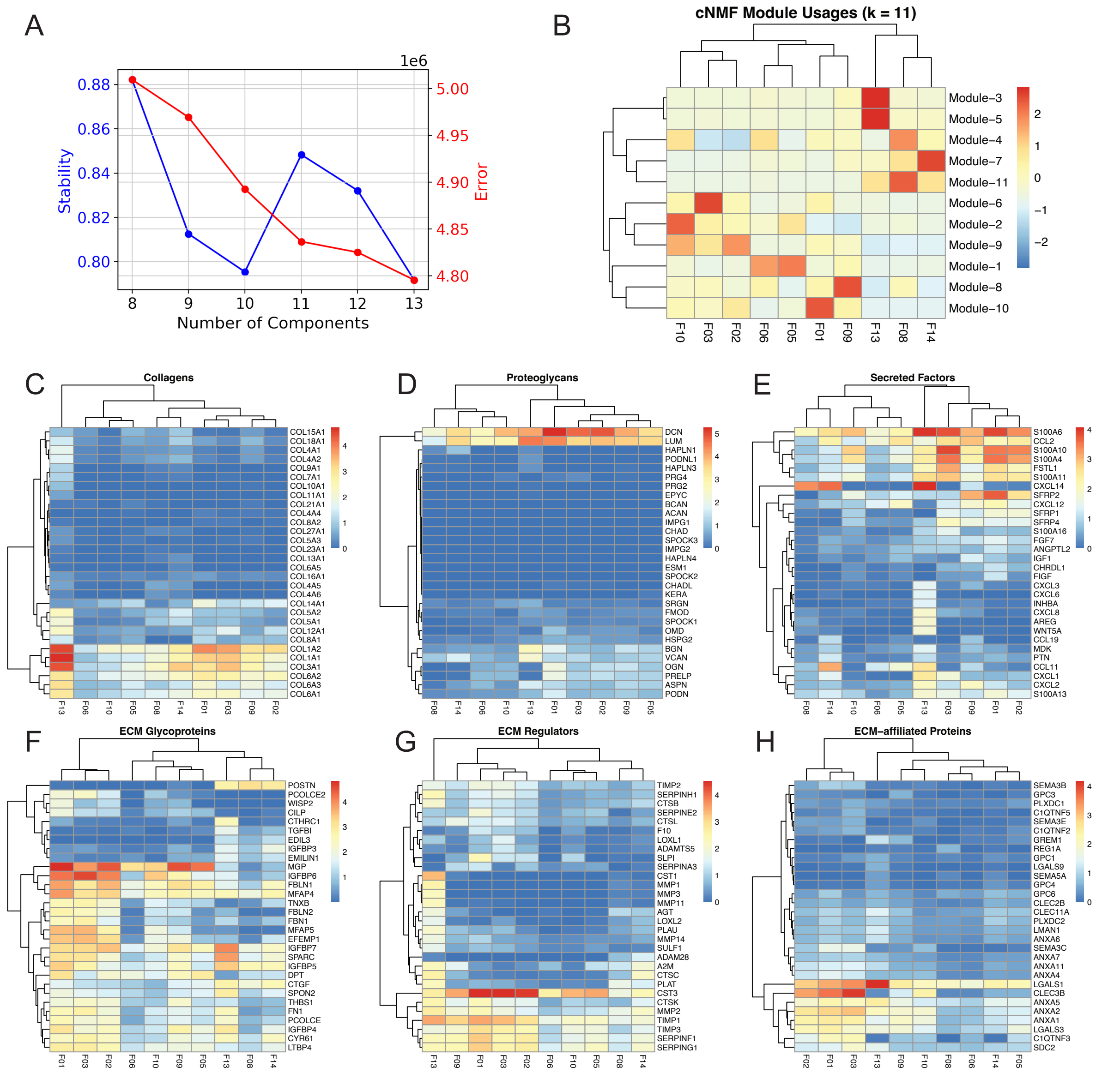

Figure S3: cNMF analysis and expression of ECM-related genes of fibroblast subtypes.

A: k-selection plot of cNMF demonstrating 11 components being most stable and least erroneous. B: Heatmap of module usage in all fibroblast subclusters. C-H: Heatmaps of average expression of ECM-related gene sets in different fibroblast subclusters. For gene sets containing more than 30 genes, the top 30 differentially expressed genes across subclusters (ANOVA) are shown.

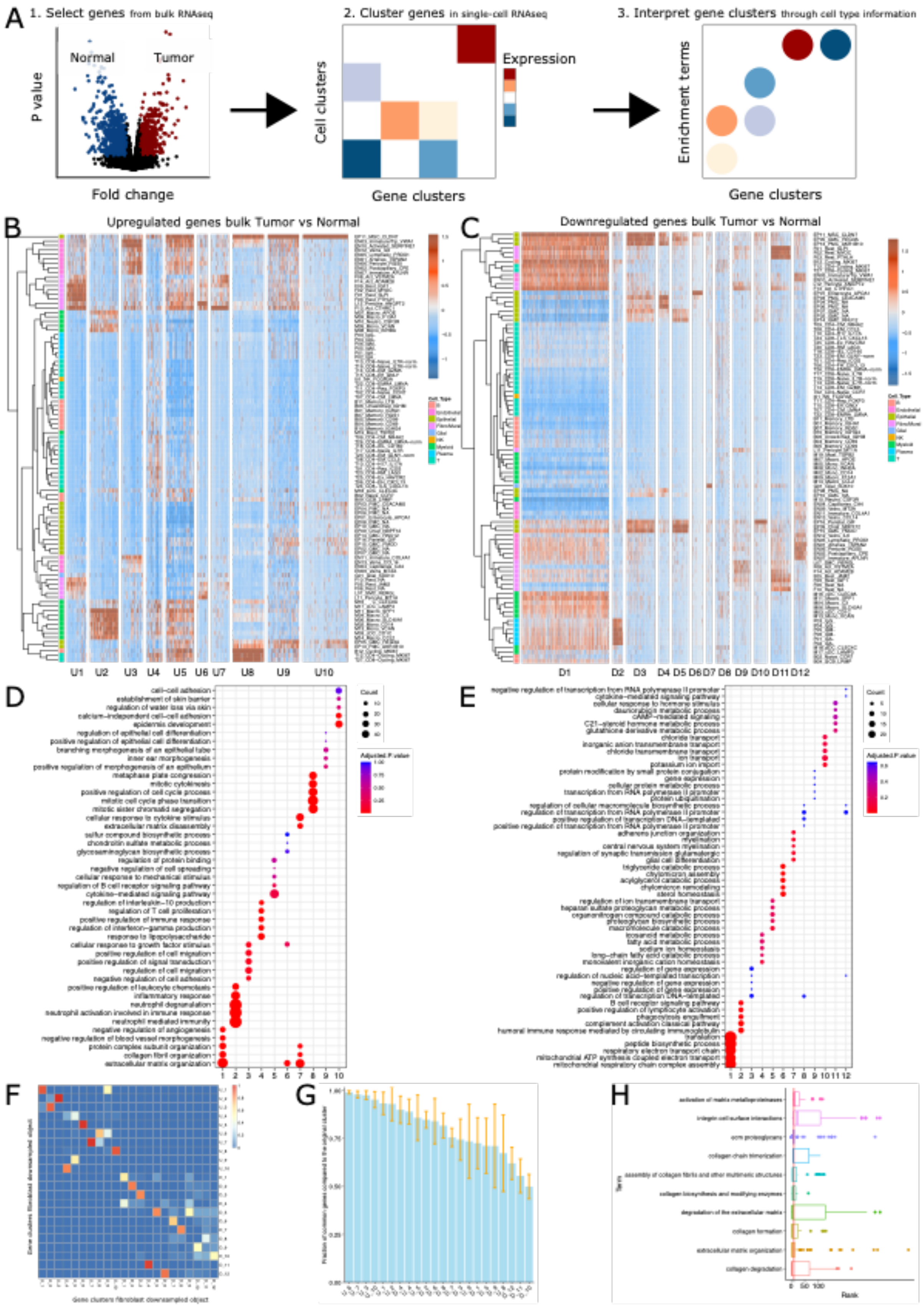

Figure S4: Bulk and single-cell RNA-seq integration.

A: Method overview of bulk and single-cell integration. From differential expression analysis between matched tumor and normal samples from 24 gastric cancer patients of the bulk RNA-seq data, 787 upregulated and 1467 downregulated genes were selected. Those genes were separately selected and clustered on the 81 subtypes of the scRNA-seq data. Resulting gene clusters were then interpreted through gene set enrichment. B-C: Genes differentially upregulated (B) and downregulated (C) from RNA-seq of malignant and non-malignant gastric cancer patients were selected and clustered as scaled average expression per minor cell type. D-E: Gene ontology analysis of gene clusters from B-C, top 5 terms per cluster are shown. F-H: Comparison of gene cluster labels between the original full dataset and a downsampled version with fibroblast reduced to 10% of the original fibroblast number. 100 random samplings performed without replacement with a fraction of genes found in the original clusters quantifying stability. One representative example shown in F, full barplot with average fraction and standard deviation shown in G. H: Gene set enrichment (GSE) analysis, rank shown of top 10 ranked GSE terms from original cluster U7 in the most similar gene cluster to U7 in the downsampled dataset.

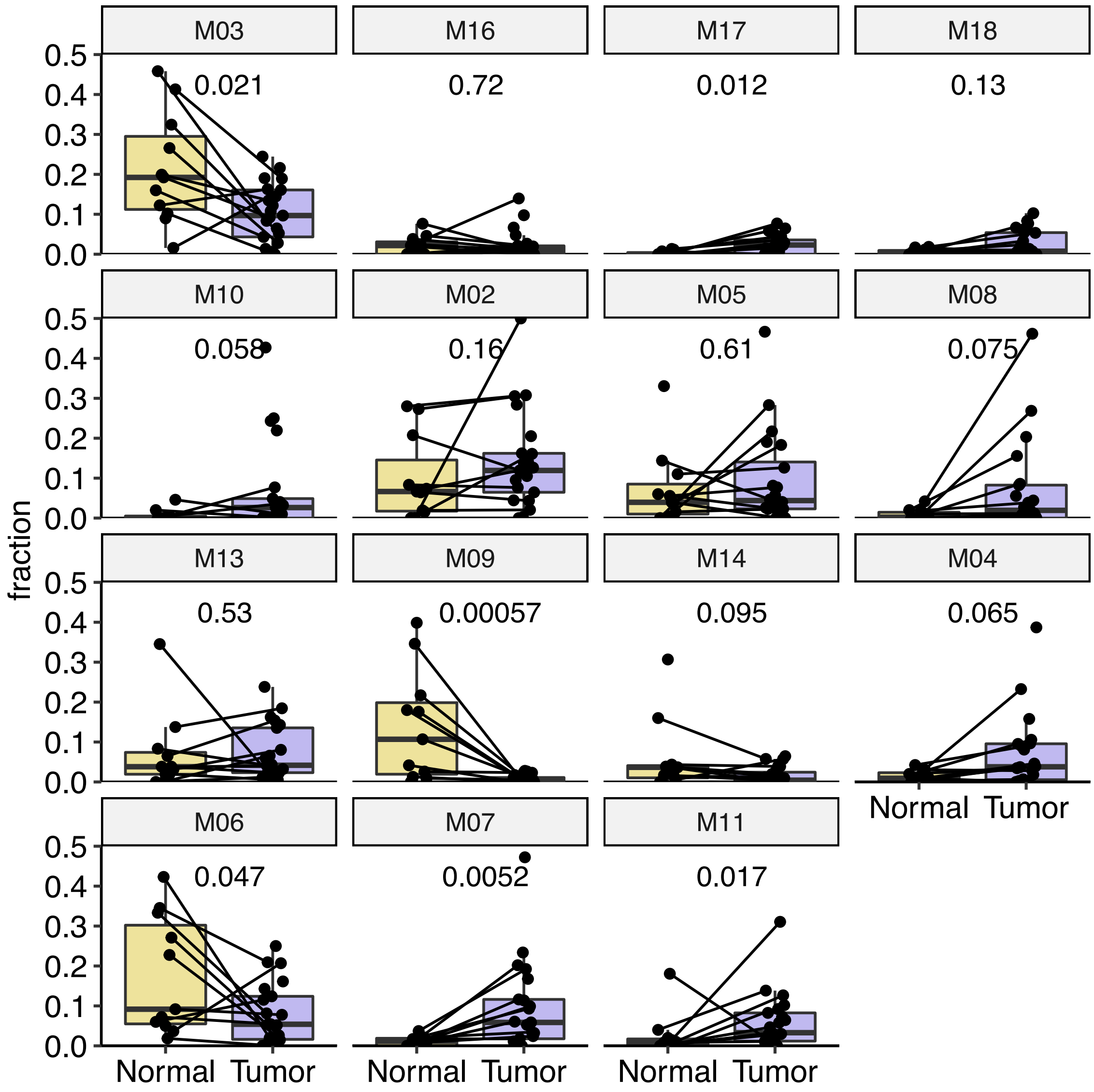

Figure S5: Myeloid cluster abundance in malignant and non-malignant stomach.

Pairwise analysis of myeloid cell cluster fraction per patient in normal and tumor tissue. Wilcoxon rank sum test, holm-adjusted *p* values per cluster shown, samples from similar patients connected.

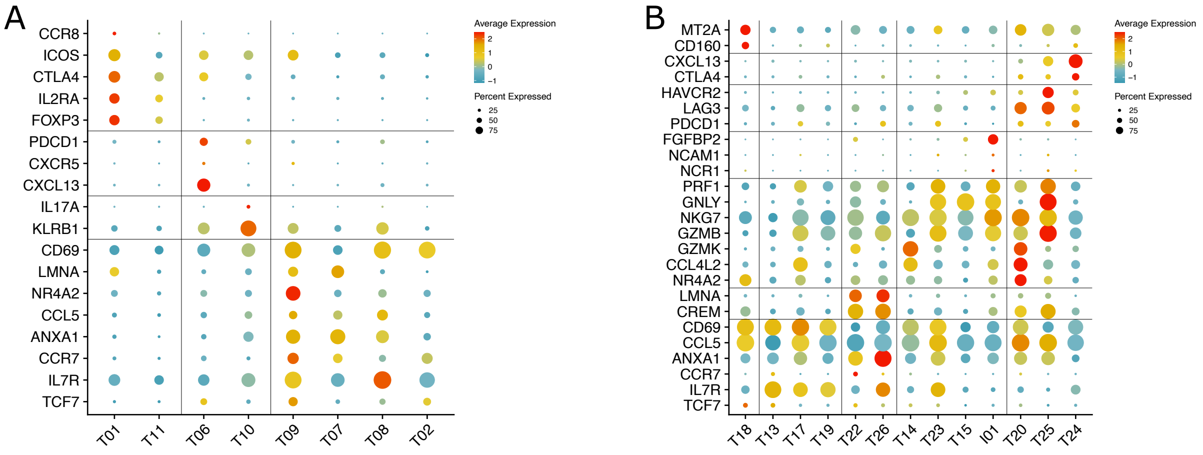

Figure S6: T cell clusters and marker genes, related to figure 5.

Dotplot showing the scaled average expression and the percentage of expression of top marker genes per cluster for CD4+ T cells (A) and CD8+ T cells (B).

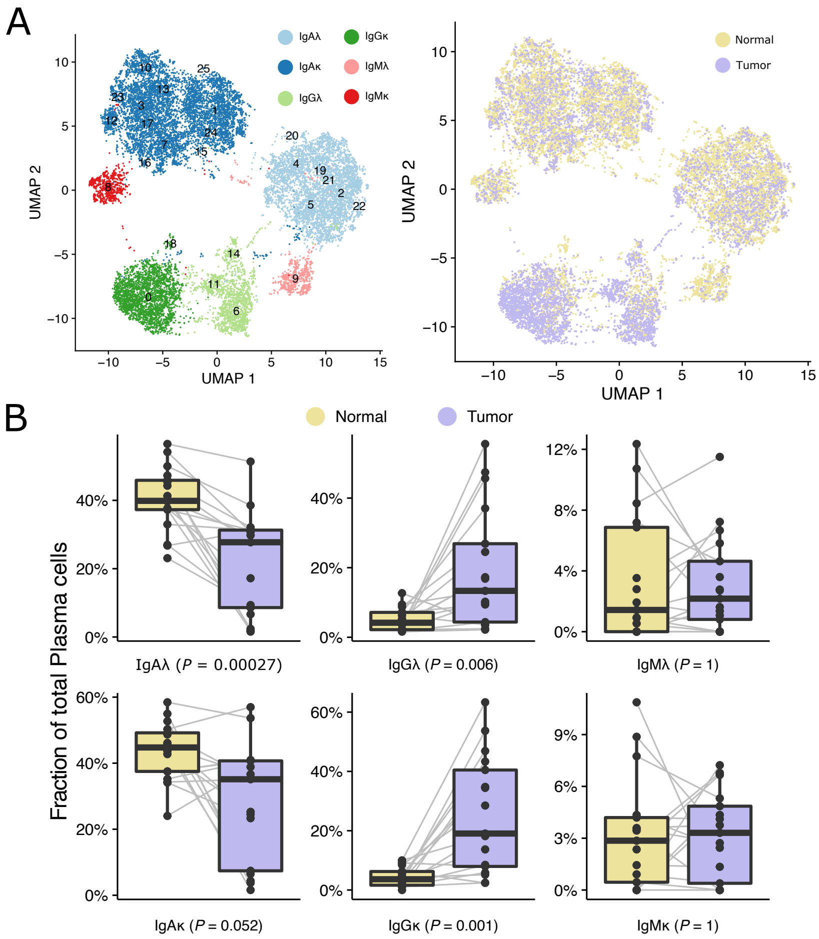

Figure S7: Plasma cells in gastric cancer

A: UMAP of 16,883 Plasma cells color coded for cluster annotation (left) and tissue (right). B: Pairwise analysis of plasma cell cluster fraction per patient in normal and tumor tissue. Wilcoxon rank sum test, holm-adjusted p values per cluster shown, samples from similar patients connected.

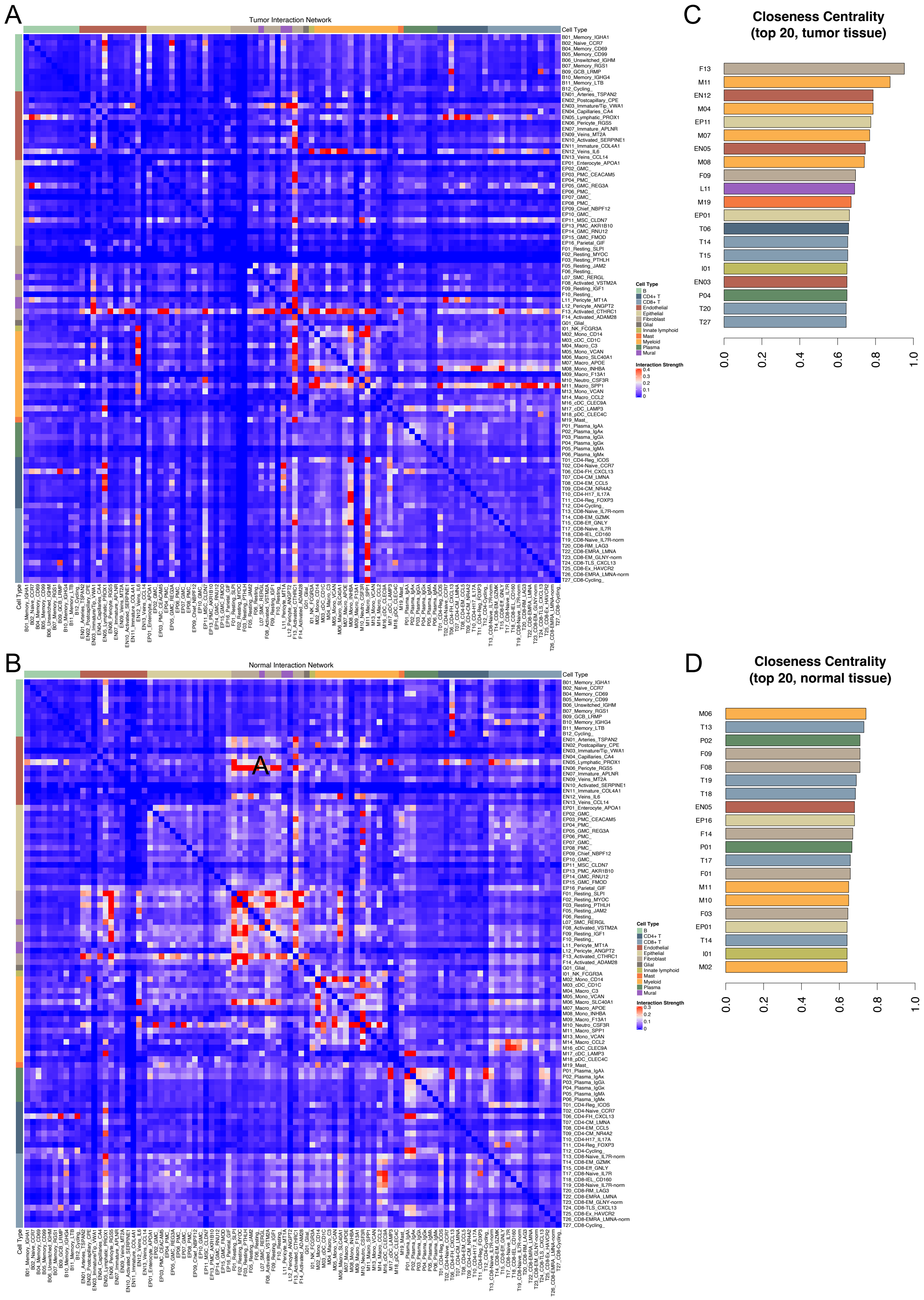

Figure S8: Different communication models of cell clusters in normal stomach and gastric cancer.

A: Connectivity heatmap of the cell-interaction network constructed using cells from the tumor tissue only. B: Connectivity heatmap of the cell-interaction network constructed using cells from the normal tissue only. Color intensity represents the connectivity of cross-cluster communications (methods). C: Closeness centrality ranking of all cell subtypes in the inferred cluster-wise cell-communication network of tumor tissue, top 10 subtypes are shown; D: Closeness centrality ranking of all cell subtypes in the inferred cluster-wise cell-communication network of normal tissue, top 10 subtypes are shown.

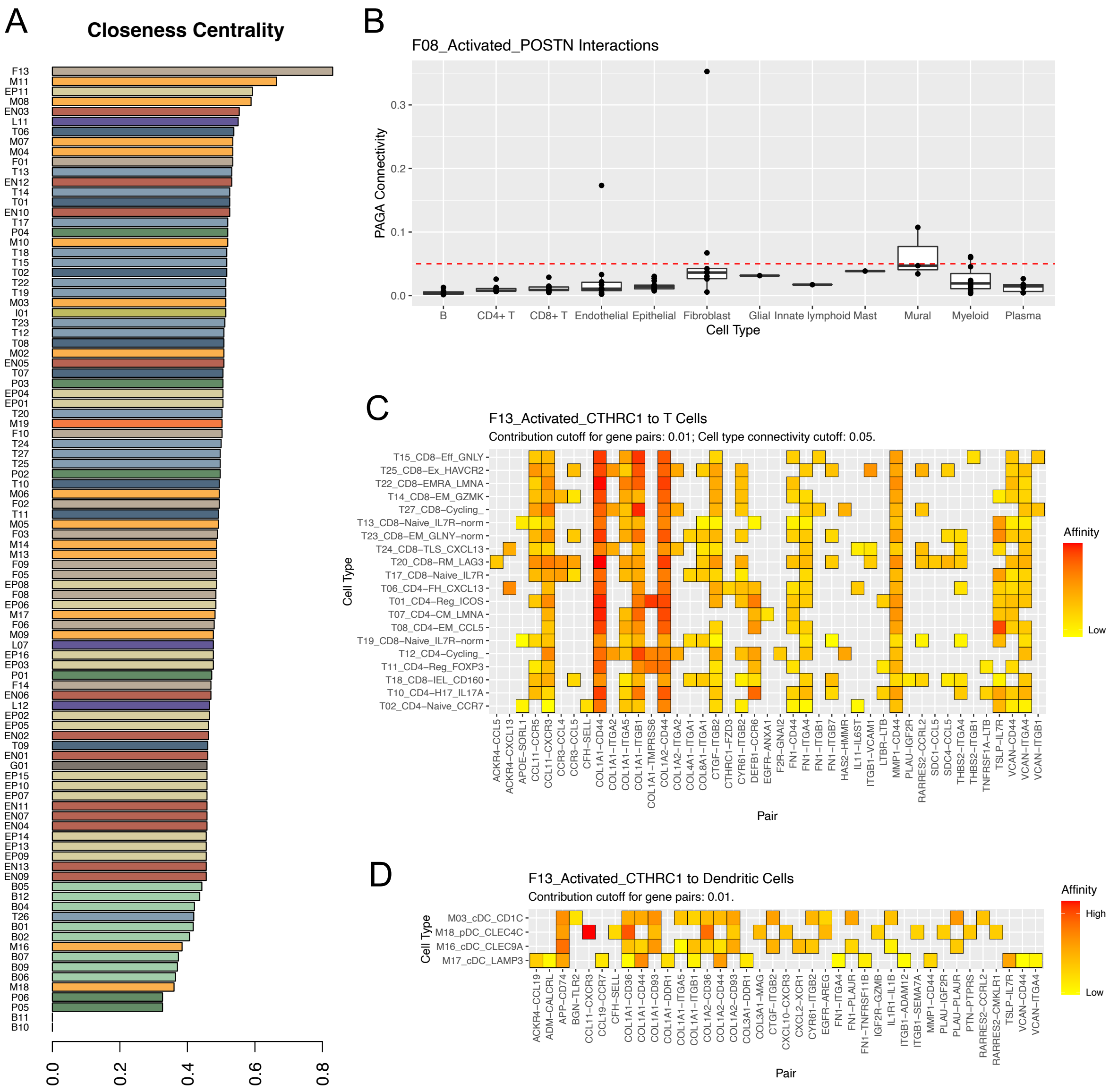

Figure S9: F13-*CTHRC1* as a central hub for interaction in the gastric tumor microenvironment. A: Closeness centrality ranking of all cell clusters in the cell-communication network constructed based on ligand and receptor expression information. Each cell cluster is treated as a node while cross-cluster communications with connectivity larger than 0.05 are treated as valid edges (methods). B: Communication strength of F08-Activated-*POSTN* with other cell subtypes, red line denotes the cluster-wise connectivity cutoff of 0.05 (methods). C: Significant Ligand-Receptor pairs in F13-Act-CTHRC1 communication with T cell subtypes. D: Significant Ligand-Receptor pairs in F13-Act-CTHRC1

communication with dendritic cell subtypes. The X-axis represent ligand and receptor pairs, with the first gene expressed on F13-Act-CTHRC1 cells and second gene expressed on the interacting cell types denoted in the Y-axis.

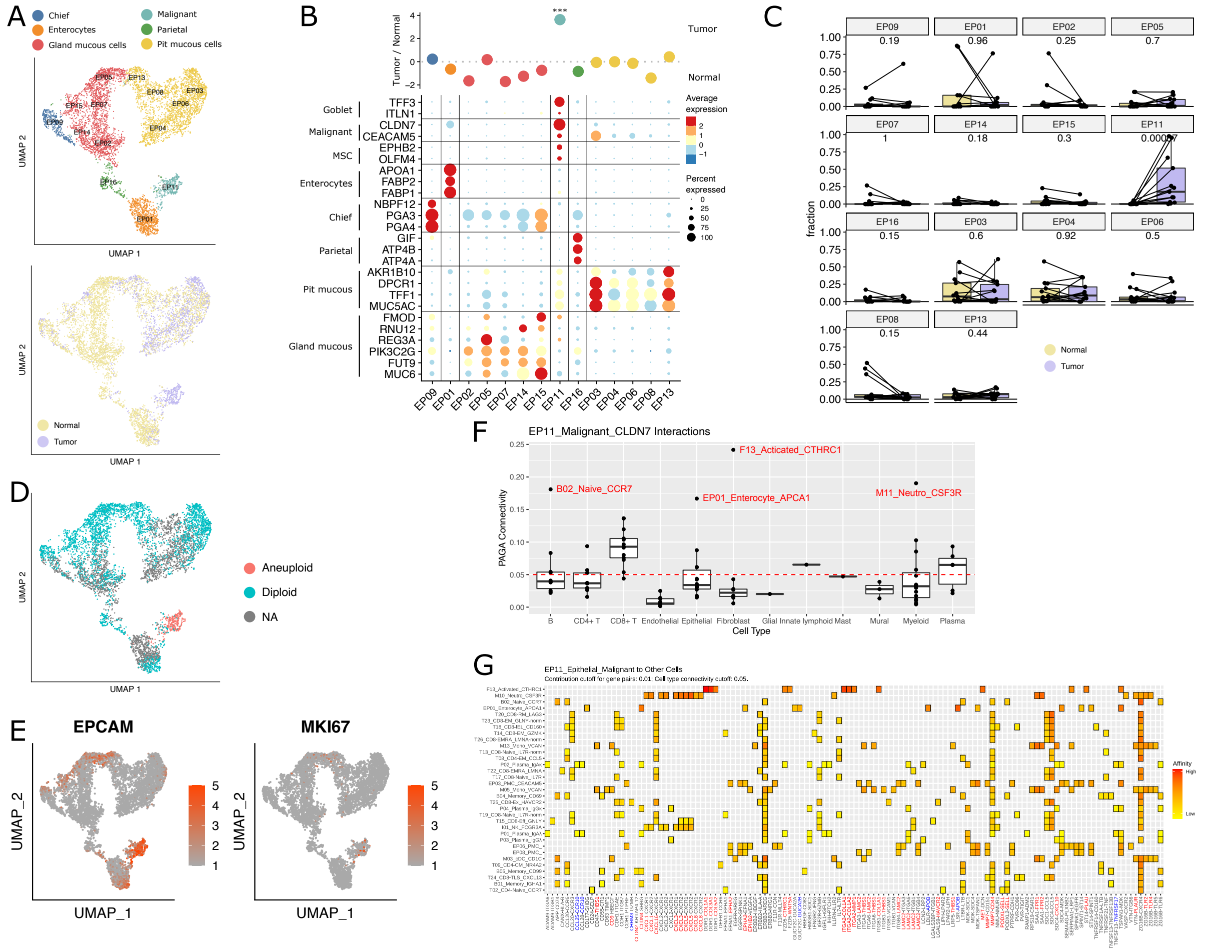

Figure S10: Epithelial cells in malignant and non-malignant stomach samples.

A: UMAP of 7,210 epithelial cells color coded for cluster annotation (top) and tissue (bottom). B: Dotplot showing the scaled average expression and the percentage of expression of top markers per cluster (bottom) and fold changes between tumor and normal (top). C: Relative fraction of epithelial clusters per sample, every dot resembles one patient, matched patients are connected. Wilcoxon rank-sum test with holm correction. D-E: UMAP epithelial cells color coded for euploidy or aneuploidy annotation (D) and expression of *EPCAM* as log-normalized counts (E), every dot represents a single cell. F: Communication strength of EP11_Malignant_CLDN7 with other cell subtypes, red line denotes the cluster-wise connectivity cutoff of 0.05 (methods). G: Significant Ligand-Receptor pairs in EP11_Malignant_CLDN7 communication with other cell types. The X-axis represent ligand and receptor pairs, with the first gene expressed on EP11_Malignant_CLDN7 cells and second gene

expressed on the interacting cell types denoted in the Y-axis. Red: upregulated gene in tumor bulk RNA-seq, Blue: downregulated in tumor bulk.

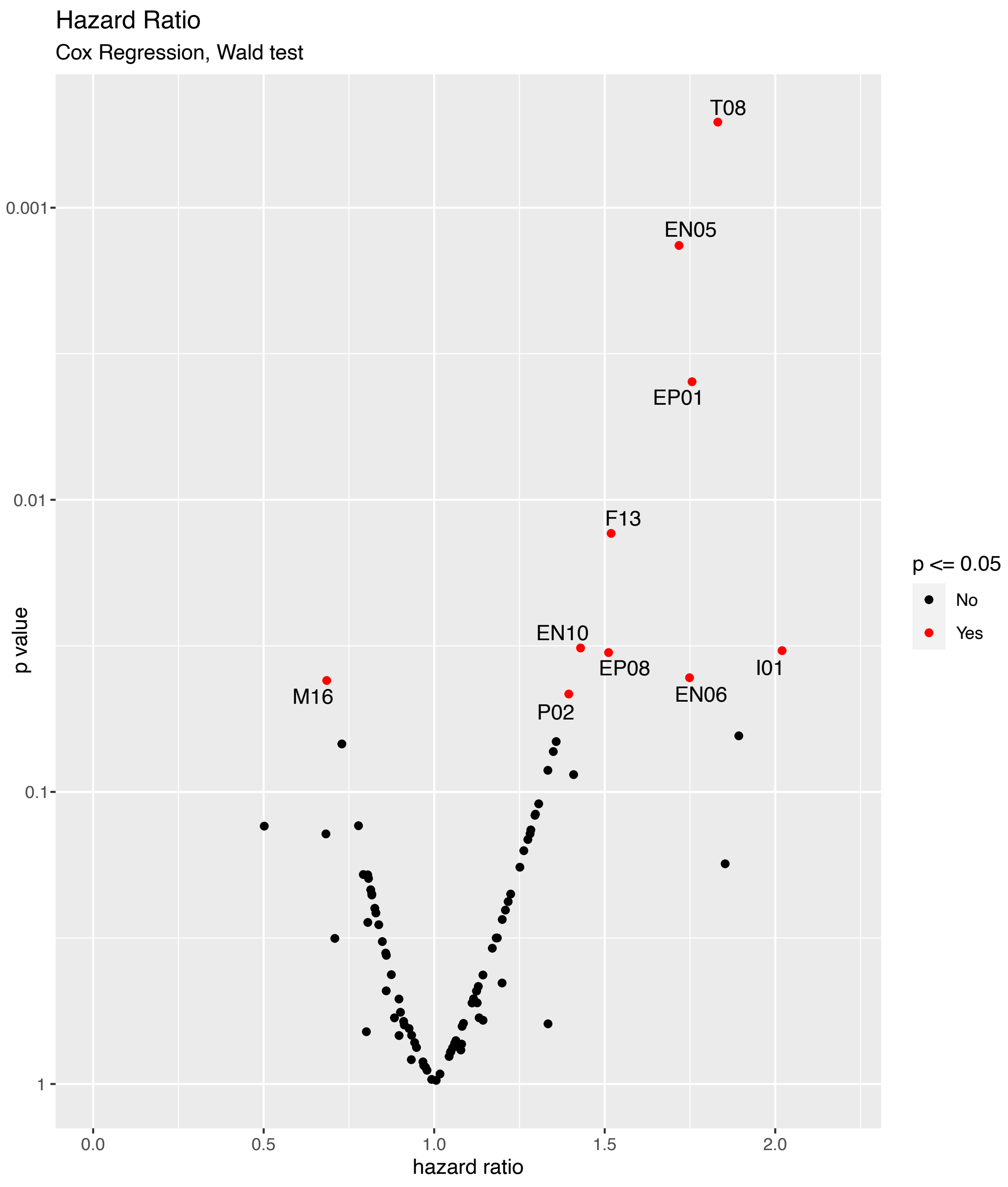

Figure S11: Volcano plot of all hazard ratios for every cell subtype deconvoluted from STAD TCGA cohort (methods). Cox regression with Wald test, significant subtypes in red (p <= 0.05).

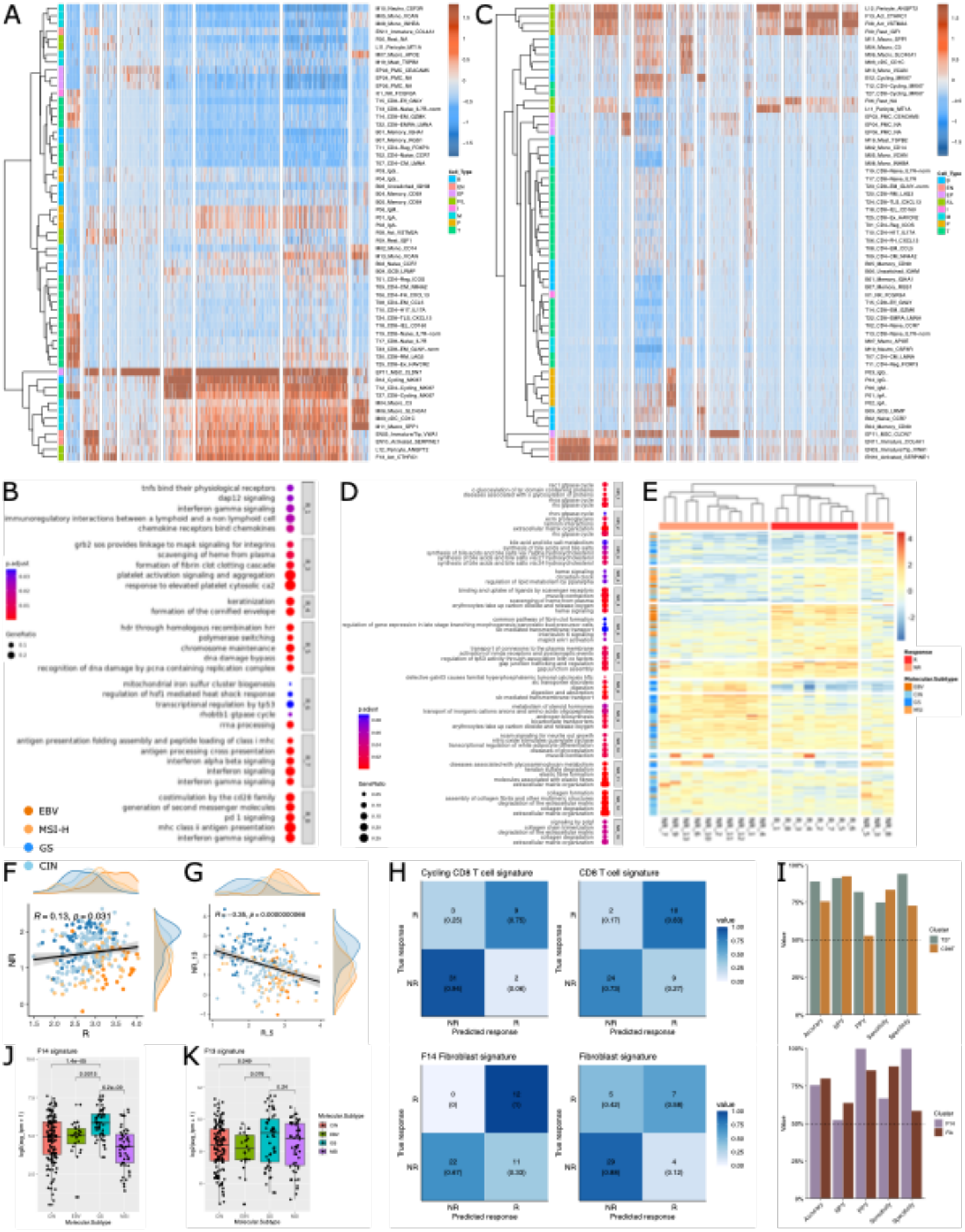

Figure S12: Cellular origin of response and non-response genes to cancer immunotherapy, related to Fig. 8.

A-B: Genes differentially upregulated (A) and downregulated (B) from RNA-seq of responder and non-responder gastric cancer patients were selected and clustered as scaled average expression per minor cell type. C-D: Reactome pathway enrichment for responder (C) and non-responder (D) gene clusters. E-G: Analysis of gene clusters in stomach adenocarcinoma cohort (n = 295) from TCGA. Heatmap with scale represented as log average expression of gene clusters (E). Average score per patient of non-response (NR) and response (R) clusters (F) and NR13 and R5 gene cluster (G). In F-G, pearson correlation. H: Confusion matrix showing predicted outcomes corresponding to the T27 cycling CD8 T cell signature and CD8 T cells signature (top) as well as the F14 fibroblast signature and fibroblast signature (bottom) on a gastric cancer immunotherapy cohort. I: Performance measurements of the gene signatures from T27 cycling CD8 T cells, pan-CD8 T cells, F14 fibroblasts and pan-fibroblasts. Shown are accuracy, negative predictive value (NPV), positive predictive value (PPV), sensitivity and specificity. J-K: Boxplot visualizing gene signature of F14 (J) and F13 (K), from Fig. 8, in TCGA STAD cohort. Every dot is one patient and patients are grouped for molecular subtype. Wilcoxon ranked-sum test.

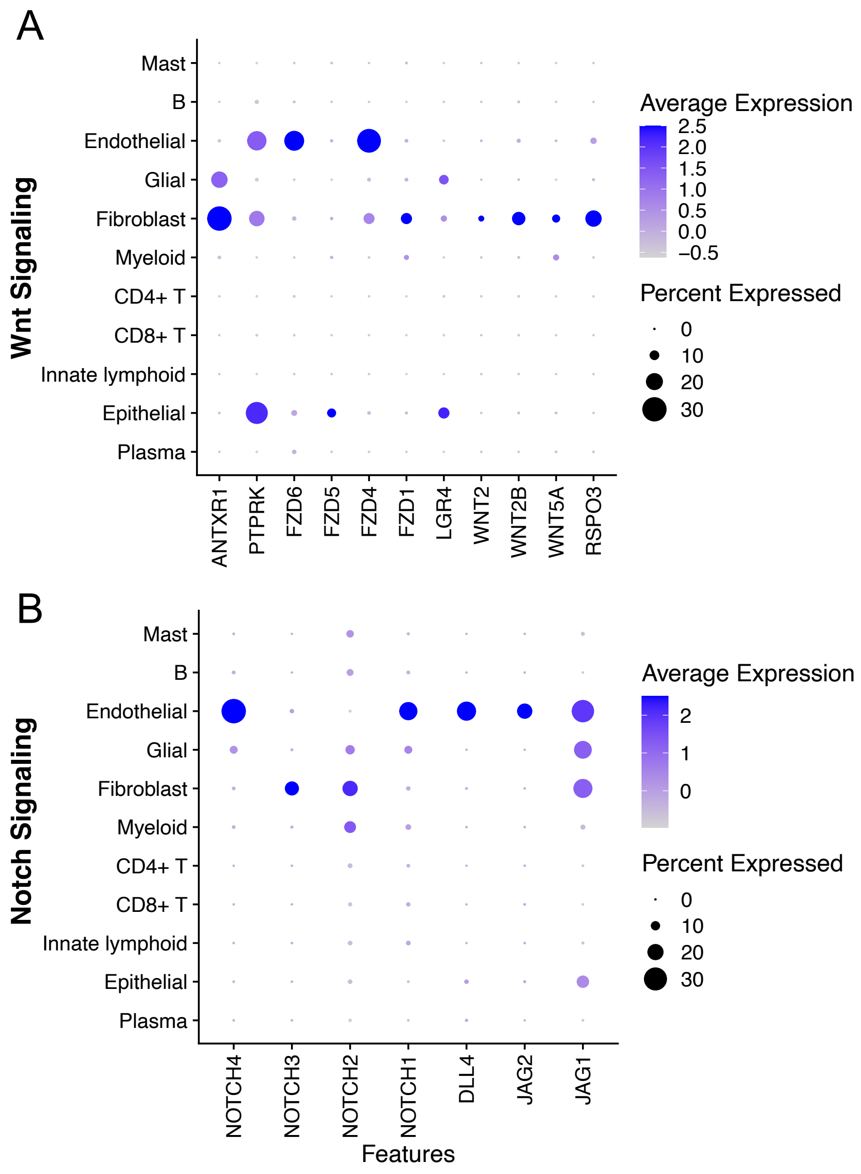

Figure S13: Expression of Wnt and Notch signaling genes in major cell types.

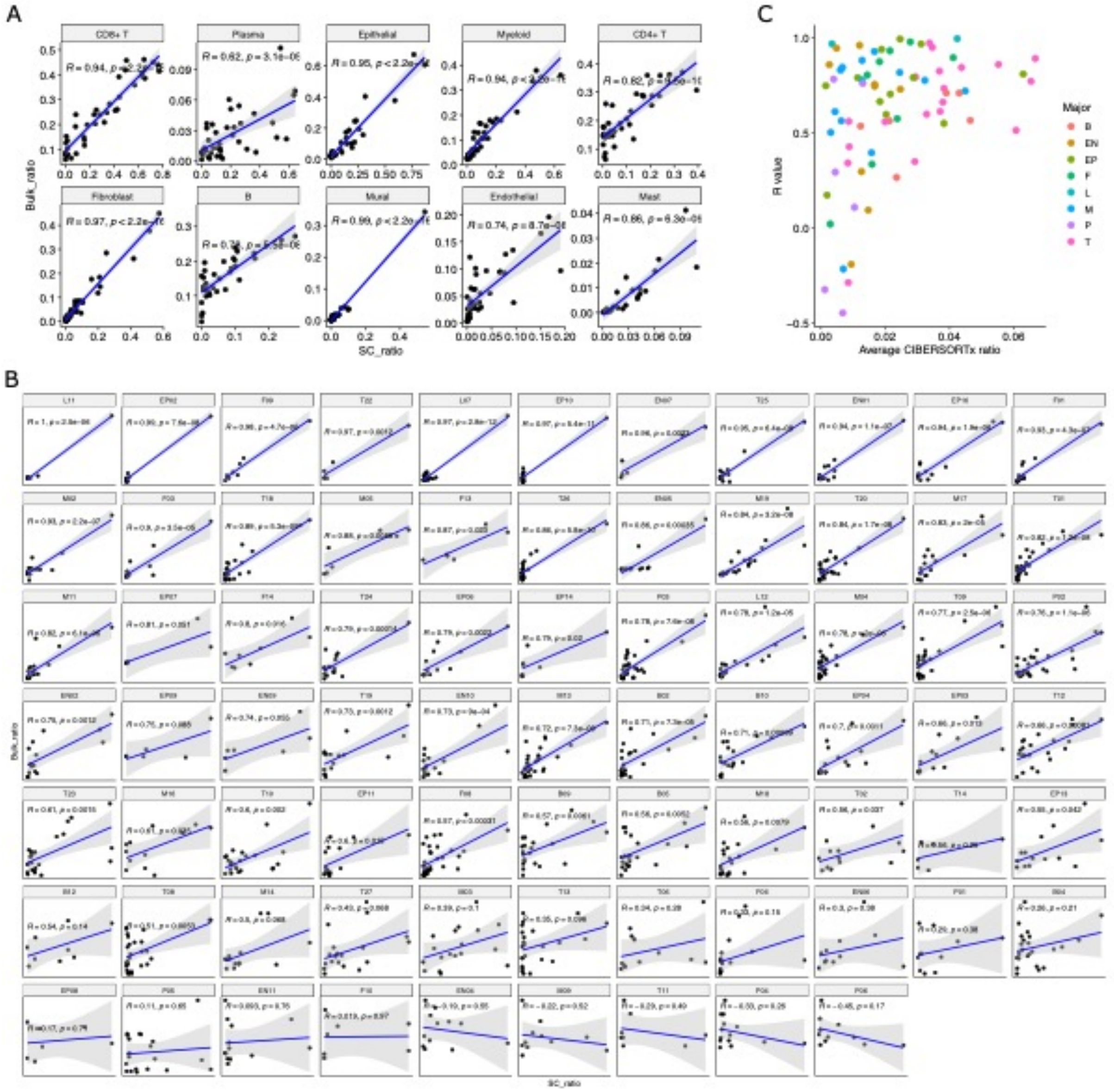

Figure S14: CIBERSORTx performance on major and minor cell subtypes.

Relative abundance levels of major (A) and minor (B) cell types in single-cell object (SC_ratio) and after CIBSERSORTx deconvolution of pseudobulk (Bulk_ratio). Comparison of average CIBERSORTx ratio per minor cell type and R value in C. Cell types colored by major cell annotation.
