# Supplementary material for: Parallel single-cell and bulk transcriptome analyses reveal key features of the gastric tumor microenvironment
Source: Genome Biol. 2022 Dec 22;23:265. doi: 10.1186/s13059-022-02828-2 (PMC9773611; doi:10.1186/s13059-022-02828-2)
Supplement: Supplementary file 8 — Additional file 8. Review history. [file 13059_2022_2828_MOESM8_ESM.docx]

Review History

**First round of review**

**Reviewer 1**

**Were you able to assess all statistics in the manuscript, including the appropriateness of statistical tests used?**

Yes, and I have assessed the statistics in my report.

**Comments to author:**

This manuscript described 96 tumor microenvironment (TME) cell types in gastric cancer from 96,623 non-epithelial single cell transcriptome data of 24 patients. Each TME cells types were tested for the correlation to the patient survival in the Cancer Genome Atlas (TCGA) data. Authors proposed the poor survival of patients with specific TME cell types such as F13-CTHRC1 and EN10-SERPINE1 from TCGA data analysis. In the analysis of bulk RNA-seq data from immunotherapy-response cohort of gastric cancer, they wanted to validate the correlation of signatures from specific cell types to the clinical response to immune checkpoint inhibitor therapy. Comprehensive analysis on TME cell types of gastric cancer will provide invaluable resources and reference to other scientists to understand gastric cancer biology. While we need more steps for the validation of those cell type markers, they proposed the potential of new biomarkers for the prediction of patient response to immunotherapy.

Major points

1. Authors analyzed single cell profiles of non-epithelial TME cells in treatment naive patients, which should be different from the TME states of patients with immunotherapy (e.g. clinical stage and history of treatment). Is the TME of treatment naive patients similar to that of immunotherapy patients?

2. Authors used non-epithelial cells for cell type identification and composed cell interaction network. However, bulk tumor tissue contains more than 70% of tumor epithelial cells and they play a central role in cell-to-cell interactions. In this sense, it is not complete cellular interaction map for gastric cancer.

3. Based on the cell type-specific markers, bulk RNA-seq data was deconvoluted to understand the enrichment of specific cell types. How deep is it possible to identify minor or rare cell types? It will help us to convince the accuracy of signature analysis of bulk RNA-seq data.

4. Is SERPINE1 expressed in EN10 only? How can we interpret the correlation of SERPINE1 expression and patient survival in terms of angiogenesis?

5. Is there any unique features of non-inflammatory macrophages in gastric cancer? Is it differentiated from tissue-resident macrophages?

6. We know the difference in pathogenesis and subtype of gastric cancer in Asia compared to that of Caucasians in TCGA dataset. Is there any potential of the difference in TME between different ethnicity?

**Reviewer 2**

**Were you able to assess all statistics in the manuscript, including the appropriateness of statistical tests used?**

Yes, and I have assessed the statistics in my report.

**Comments to author:**

Summary and General Comments

The authors provide an extensive resource that integrates matching tumour-normal datasets from both single-cell RNA-Seq, bulk transcriptomics and exome-sequencing. The analysis is clearly described and will surely be a valuable clinical research resource in understanding the role of immunotherapy on the TME in gastric cancers. The focus of the study is on the non-epithelial compartment of the gastric cancer TME, from which the authors identify a remarkable set of 96 cell-types. Of these, a number have also been shown to play a role in immunotherapy response in other solid cancers, highlighting the benefit of carrying out detailed integrated cancer studies, such as this, to understand the impact of cellular heterogeneity on important clinical problems. Finally, using cell-type deconvolution the authors show correlations between their identified tumour cell-types and treatment response and develop cell-type based classifiers to identify TME based markers of ICI response.

Main Comments

* A key strength of this resource is the large number of matched tumour-normal pairs. However, it's not clear how the tumour vs normal comparisons were carried out and how the robustness of the comparisons should be assessed. The study would benefit from more detailed quantification, statistics and discussion on the power of the dataset to make these comparisons. For example, on pg 6 they identify drastic reductions of resting fibroblasts in the gastric TME. Can the authors please describe how these comparisons were carried out and what statistics were used to quantify the differences? In particular, how were the potential confounding effects of differences in cell-numbers from the normal and tumour datasets taken into account?

* Similarly, a greater discussion of inter-sample differences would be beneficial. For example, I note that batch integration methods and HSP-based gene signature regression have been used, but no discussion is made as to the cell cluster composition in terms of the number of different tumours (i.e., whether some are dominated by cells from a single/minority of tumours). I note that this has been shown visually, via UMAP in fig S1E, but some more detailed discussion and quantitation should be given to complement this. This is particularly important as the authors identify 96 cell-types, which is a large number and therefore it is useful to understand which of these are potentially found in only a small subset of tumours.

* As part of the focus on non-epithelial cells in the TME, EPCAM+ cells are depleted prior to single-cell capture. However, a number of points arise from this process:

1. Even after depleting cells of non-epithelial origin, a substantial number of epi cells seem to remain (fig 1b). Could the authors comment on significance of these cells and whether they have been seen in other studies of gastric cancer. For instance, are these neoplastic cells or also found in the matching normal tissue? Do they express EPCAM in the single-cell data?

2. Also, these cells are clearly playing a role in the TME and are found to be well-connected/enriched in a number of analyses (e.g., the cell-cell communication networks) again warranting further discussion.

3. I wonder what effect the depletion of EPCAM+ epithelial cells has on the biclustering based approach and resultant identification of response and non-response groups (e.g., as shown in fig 8 and S9). For instance cluster R4 is strongly enriched for epithelial properties. Could the authors comment on this?

4. I think that the term "comprehensive" (conclusion, line 29) is not quite correct for this study as the representation of the epithelial cell compartment is incomplete due to EPCAM+ cell depletion. I suggest re-phrasing this.

5. Also, (on pg 12, lines 52-53) when stating that "non-responder gene programs were associated with stromal, plasma, and epithelial cells …" it's unclear which epithelial cells are being referred to here, i.e., are these the EPCAM- cells in the single-cell dataset? I think this needs clarifying.

* As part of the cell-communication network analysis the authors note that "communication models in tumour and normal tissue differed significantly (Fig. S6)". This is not immediately clear to me. I think a more quantitative justification for this conclusion is needed. Also, the colours of the cell-types are different in the rows and columns of the matrix shown in this figure. This is confusing and I think needs a clearer figure legend.

* The authors use the cell-types identified in the TME to generate binary classifiers to predict contribution to ICI response. This is done in the form of a series of AUROCs. However, it's difficult to appreciate the significance/impact of the difference between, for example, an AUROC of 0.85 (cycling CD8+ T-cells) and 0.81 (regular CD8+ T-cells). Is there any way to further quantify these differences? Further, it would be useful to see the full list of AUROC scores for all 96 cell-types as a supplemental figure or table to get an appreciation of the variability of performance for each celltype.

Data processing and availability

* Pg14 line 5: notes that 117506 "post-filter" cells were identified, however, in the results on pg5 only 96,623 cells were used in the clustering. I assume this is related to the "low-quality" high mitochondrial cluster that was removed (pg 14/Fig S1d)? Could the authors please clarify this.

* This will be a valuable resource to the community and therefore it's vital that data and analysis code be made available upon publication. As a minimum all processed results and analysis scripts should be made publicly available (e.g., in GEO and github), with raw data available through appropriate access-controlled channels. I also suggest adding the single-cell data to a repository such as the Broad single-cell explorer. These steps will ensure reproducibility and much increase the audience and accessibility of the resulting dataset.

MINOR

* Pg6, lines 54-56: "In addition, the RNA expression of these genes is upregulated in tumors from many different indications in the cancer genome atlas (TCGA) cohort (Fig S2d).". This figure only shows the gene CTHRC1, whereas the text indicates that it should show more genes?

* Pg7, lines 9-10: "Upon analyzing the 3,684 endothelial cells in our data, we found eight clusters that corresponded to six different endothelial cell subtypes (Fig. 3a).". The cluster labels are difficult to read, but I think that there are actually 9 EN cluster shown? Also, the numbering is confusing as the cluster labels indicate that there should be at least 12 clusters (i.e., EN01-EN12)? This should be clarified.

* Fig 3c: It's not clear what the criteria for selecting this particular set of genes was.

* I think that "domestic" should be replaced with "tissue resident" in the conclusion (line 25)?

**Reviewer 3**

**Were you able to assess all statistics in the manuscript, including the appropriateness of statistical tests used?**

Yes, and I have assessed the statistics in my report.

**Comments to author:**

Summary:

In this comprehensive catalogue of cell types involved in human gastric cancer, Kang and colleagues have implemented single-cell and bulk transcriptomics approaches to pinpoint cellular combinations of predictive value. Notably, they have dissected cellular contributions from fibroblasts or cancer associated fibroblasts (CAFs), endothelial, myeloid and T cell populations in malignant versus non-malignant samples, identified key sub-populations within each cell type, their cellular interactions and how this may impact survival outcomes in gastric cancer human cohorts. Overall, this study demonstrates common principles of how an immunosuppressive TME emerges, through the increase in regulatory T cells, immunosuppressive DCs, macrophage populations with an elusive anti- as well as pro-inflammatory signature and activated CAFs.

This is an important resource for the TME field and this manuscript can benefit from a few clarifications and recommendations listed below.

Major comments:

1. Can the authors provide references and/or reasoning for categorizing fibroblasts into: resting, activated and myofibroblasts? Some markers used in this study such as RGS5 mark pericytes as well - have the authors ensured exclusion of this cell type in the fibroblast cluster?

2. Fibroblast clusters described (for eg resting fibroblasts) are dictated by the choice of resolution parameters used for clustering. Can the authors comment why so many different resting fibroblasts are observed in normal adjacent tissue samples - are these distinct cell types or cell states? Applying algorithms such as cNMF [Kotliar et al., 2019 eLife] will help clarify this.

3. What are the differences in genes between U1 and U6, both implicated in ECM organization? In Figure 2C F13-CTHRC1 upregulates genes in U1 and U7 involving processes of ECM organization and destruction - can the authors explain the functional role of CTHRC1 fibroblasts given that they express genes from opposing processes? Can the authors classify various fibroblast subsets based on the ECM atlas described here: matrisome project (http://matrisomeproject.mit.edu/)?

4. In Figure 8D, it will be important to understand which resting fibroblast populations express high NR11 genes (given that this signature is restricted to resting fibroblasts)

5. Do F13-CTHRC1 express LRRC15 and is this population driven by TGF-beta signaling as well? Can the authors comment on why F14-ADAM28+ fibroblasts are more predictive of response versus non-response in the STAD cohort as opposed to F13-CTHRC1 that interact with multiple cell types in the GC single-cell atlas?

6. Have the authors considered categorizing impactful subpopulations within each cell type by tumor mutational burden or molecular subtypes - is a sub-population overrepresented in one subtype over another?

7. Can the authors show survival outcomes in the TCGA cohort for non-inflammatory and proinflammatory macrophage subpopulations (M04,M07,M11 and M08) enriched in GC as opposed to a common pro and non-inflammatory gene signature?

8. Sathe et al., 2020 Clinical Cancer Res also describe a single cell atlas of the gastric cancer TME, albeit with fewer patients. Do the authors also observe the LGR4-RSPO3 axis in fibroblasts and Notch signaling characteristic of endothelial cells? It will be worthwhile to map/project this dataset on your atlas and update current knowledge about the gastric TME ecosystem.

Minor comments:

Figure 2A: Use contrasting colors to represent tumor and non-tumor samples. It is hard to distinguish between two similar shades of green.

Figure 2C: Please clearly mention in the legend or annotate in the figure that U1, D9 etc correspond to gene sets up- or down-regulated in bulk tumor versus normal comparison.

Figure 5: Can the authors show tumor versus normal UMAP here for the readers to quickly assess where the different T cell populations are localized?

Figure 8A: Increase font size of gene names.

Figure 7 survival plots: add risk table clearly showing number of patients per group (survminer package in R)

Figure S9D and E: Please check whether the Pearson correlation is computed for panel D or E (average NR and R score per patient or NR13 versus R5. Presumably, this is for E). Could the authors also add a legend for molecular subtypes for D and E (in addition to the one in C)?

Please use consistent nomenclature: anti-inflammatory macrophage as opposed to non-inflammatory.

Grammar: check for usage of where and were.

E.g. page 11, line 60: "Activated endothelial subtypes have been documented in prostate cancer and 'where' shown to express CAF markers". 'Where' should be replaced with "were".

**Authors Response**

**Point-by-point responses to the reviewers’ comments:**

Reviewer #1

*Reply: We thank the reviewer for the very positive comments on our analyses and the significance of our work, and also greatly appreciate all the constructive suggestions and criticisms, which have helped us further improve the novelty and quality of our manuscript. Point-by-point responses to the reviewer’s comments are as follows.*

Major comments

Authors analyzed single cell profiles of non-epithelial TME cells in treatment naive patients, which should be different from the TME states of patients with immunotherapy (e.g. clinical stage and history of treatment). Is the TME of treatment naive patients similar to that of immunotherapy patients?

Reply 1:

*Dear Reviewer, the data from Kim et al. is also derived from immunotherapy treatment naïve patient samples. In general, it would be highly interesting to see what changes occur in the TME with immunotherapy treatment but to the best of our knowledge we are not aware of post-treatment samples from gastric cancer patients that could be used to perform such an analysis. As has been shown in melanoma 1, glioblastoma 2 and breast cancer 3, the TME of different tumor types and stages contains the same cell subtypes pre- and post-treatment (albeit with different abundance). Therefore, we do not expect post-treatment gastric tumor samples to harbor cell types that are not identified in pre-treatment samples. Our bi-clustering approach works by mapping the differentially expressed genes from bulk samples to all main- and sub-cell types (which should exist also in post-treatment samples) identified in the single cell analysis. As will be discussed in more detail in reply 9.3, our method is robust against changes in cell type abundance (as might occur due to immunotherapy treatment). We therefore do not expect the results of our bi-clustering analysis to be significantly affected by the treatment status of the single cell analysis samples.*

Authors used non-epithelial cells for cell type identification and composed cell interaction network. However, bulk tumor tissue contains more than 70% of tumor epithelial cells and they play a central role in cell-to-cell interactions. In this sense, it is not complete cellular interaction map for gastric cancer.

*Reply 2:*

*We thank the reviewer for highlighting the epithelial cell contributions to the interaction network present in gastric cancer. In fact, we had included the epithelial cells contained in our data in all the analyses (see Figure 6), however, since the manuscript is focused on the TME we had not discussed their contribution in the previous version of the manuscript. We have now added a paragraph describing the function of epithelial cells and their role in the interaction network into the manuscript from page 12 line 6 to page 13 line 5.*

*Due to our strategy of enriching for EPCAM negative cells, our single cell data contains fewer EPCAM positive cells than would normally be the case. In principle this could affect the interaction network. However, the PAGA algorithm used by us to quantify cell-cell communications is robust against the relative number of cells from each cell type in a dataset. We confirm this below by sampling each population down to 1000 cells and repeating the cell communication analysis using the same parameters. As is shown in the figure, we observed the same four cell types (F13, M11, M08, and EP11) as the most central cell subtypes in the interaction network.*

Based on the cell type-specific markers, bulk RNA-seq data was deconvoluted to understand the enrichment of specific cell types. How deep is it possible to identify minor or rare cell types? It will help us to convince the accuracy of signature analysis of bulk RNA-seq data.

*Reply 3:*

*Bulk RNA-seq deconvolution has been shown to be precise for major cell populations but so far it has not been clear to what detail cell subtype abundance can be predicted from bulk RNA-seq data 4. To have an idea about the accuracy of the deconvolution based on our 81 cell subtypes we created a pseudobulk matrix from our single-cell data by summing up all read counts in each sample and then applied again CIBERSORTx for deconvolution.*

*We first checked how well the CIBERSORTx predictions for our 10 major cell types correlate with the actual single-cell fractions in our study. All cell types showed highly significant Pearson correlations of r~0.8 or above (panel A below) between predictions and actual cell type frequencies. We then investigated the prediction accuracy of CIBERSORTx for the 81 cell subtypes. As shown in panel B, results were in significant agreement with the preselected single-cell abundances for most of the cell subtypes. In fact, over 80% of the cell-subtype frequencies predicted by CIBERSORTx had a Pearson correlation higher than 0.5 with the actual data. These include the cell subtypes F13, EN10 and M16 which we highlight in the paper. For these cell types, Pearson correlation coefficients of 0.86, 0.74, and 0.59 were obtained, respectively (panel B). Only 18 cell types had correlation coefficients below 0.5. We noticed that these cell types either had very low abundance close to the detection limit (panel C) or possessed only a few specific marker genes as is the case for some plasma cell types. We believe that the accuracy of these results demonstrates that marker gene-based deconvolution via CIBERSORTx can successfully be used to also deconvolute the presence of most cell subtypes in bulk RNA-seq data. We have included this data as supplementary figure S14 and added a paragraph in the methods section at page 25 line 3-18.*

Is SERPINE1 expressed in EN10 only?

*Reply 4.1:*

*As shown in the panel below (and Fig 3b in the manuscript), SERPINE1 is most highly expressed in EN10 but is also found in the cluster EN03, albeit at lower levels. Both these cell types are themselves highly enriched in tumor samples (Fig. 3b and below).*

How can we interpret the correlation of SERPINE1 expression and patient survival in terms of angiogenesis?

*Reply 4.2:*

*To the best of our knowledge, EN03 and EN10 endothelial cells (ECs) have not been described in gastric cancer before and only to a limited extent in other cancer indications 5. Our annotation of the EC clusters is based on the extensive work of Goveia et al. 5 who developed a single-cell atlas of tumor ECs. By using their marker genes (APLNT, VWA1, HSPG2) we believe EN03 and EN10 to belong to the group of immature stalk cells. In addition, EN03 also expresses markers of tip cells such as ESM1, PGF, CXCR4 while EN10 expresses markers including SERPINE1, PRCP, and SELE that were not described by Goveia et al. The role of these endothelial cell subtypes in angiogenesis has not been described.*

*In particular, SERPINE1 is known to function as a key component in angiogenesis with blocking of SERPINE1 leading to an inhibition of angiogenesis in malignant pleural mesothelioma 6, and overexpression of SERPINE1 promoting malignant progression in gastric cancer 7. In gastric cancer, angiogenesis is associated with an attenuated tumor microenvironment, aggressive biology, and worse survival 8. In our opinion this suggests a potential role of EN03 and especially EN10 in gastric cancer progression, with SERPINE1 as a potential mediator and drug target candidate. However, since the data is not conclusive about the function of these cells, we would suggest not to add further text to the manuscript.*

Is there any unique features of non-inflammatory macrophages in gastric cancer?

Reply 5.1:

*Macrophages in our dataset were annotated as non-inflammatory based on the expression of marker genes such as APOE 9, MAF 10, FOLR2, and TREM2 (see fig. 4 in manuscript) which have been associated with immune suppression in literature. In addition to the above markers, these cells further express for instance high levels of C1QC, C1QA, and C1QB 11. In contrast, expression of pro-inflammatory markers such as VCAN and S100 genes is excluded from these macrophage clusters. To address whether the anti-inflammatory macrophages found in gastric cancer are a unique macrophage subtype or whether they resemble macrophages found in other tumor types we compared the co-expression of several marker genes for the different macrophage subsets in our dataset with the co-expression of the same marker genes in macrophages found in data sets from several other indications. As can be seen in the panel below, the anti-inflammatory markers from our dataset (e.g., APOE and C1Q) appear to be also co-expressed in macrophages from the other indications and their presence appears to be mutually exclusive to the expression of pro-inflammatory markers such as VCAN and S100A across datasets. In contrast, pan-macrophage markers such as LYZ and CD163 are found across all macrophages in all studies.*

*From this analysis we conclude that macrophages from different TMEs form similar subtypes with possibly similar functionality. It would require integrating all datasets into one study to investigate more closely whether there exist genes (pro- or anti-inflammatory) that are uniquely expressed in macrophages from the TME of gastric cancer. While we believe that such a detailed study would be of great interest, due to the high complexity of such an endeavor, we have not performed such an analysis at this point and would suggest to not add further text about the above comparison to the manuscript.*

Is it differentiated from tissue-resident macrophages?

Reply 5.2:

*To investigate possible differences between anti-inflammatory and tissue resident macrophages, we performed differential gene expression analysis between the two cell types. As shown in the volcano plot below, we found many significantly differentially expressed genes indicating that these populations are indeed differentiated from each other. Tissue-resident macrophages for instance express significantly higher levels of FOLR2 which is specifically expressed by tissue-resident macrophages in breast cancer 12 as well as of CCL2 13, LYVE1 14, SEPP1 15 and F13A1 16 all of which have been reported as markers for tissue-resident macrophages in multiple different organs. In addition, we found two transcription factors, EGR1 and NR4A1, that could be important mediators of tissue-resident macrophage functionality. We believe that the abundant marker genes of gastric tissue-resident macrophages and the description of tissue-resident macrophages in other organs makes them substantially different from anti-inflammatory macrophages. We have now clarified the main differences between tissue-resident and anti-inflammatory macrophages in the main text of the manuscript at page 9 lines 5-8.*

We know the difference in pathogenesis and subtype of gastric cancer in Asia compared to that of Caucasians in TCGA dataset. Is there any potential of the difference in TME between different ethnicity?

*Reply 6:*

*We examined the TCGA-STAD cohort for differences in TME composition between ethnicities. Among the 312 samples with ethnicity information (74 Asian, 238 white), no significant differences in major cell type scores or subtype scores were found (t test, adjusted p value > 0.05). For the manuscript, we added “In general, we did not identify significant differences in the TME by ethnicity or tumor mutational burden” to the Discussion at page 16 lines 20-21.*

Reviewer #2

*Reply: We thank the reviewer for the very supporting comments on our study and the insightful suggestions for improvement which are highly appreciated and have now been taken into account. Point-by-point responses to the reviewer’s comments are provided below.*

Major Comments

A key strength of this resource is the large number of matched tumour-normal pairs. However, it's not clear how the tumour vs normal comparisons were carried out and how the robustness of the comparisons should be assessed. The study would benefit from more detailed quantification, statistics and discussion on the power of the dataset to make these comparisons. For example, on pg 6 they identify drastic reductions of resting fibroblasts in the gastric TME. Can the authors please describe how these comparisons were carried out and what statistics were used to quantify the differences? In particular, how were the potential confounding effects of differences in cell-numbers from the normal and tumour datasets taken into account?

*Reply 7:*

*We thank the reviewer for this comment and fully agree that the reader will appreciate a more detailed explanation of the applied statistics. To describe our approach, which has also been described and frequently used in other scRNA-seq research papers 17–20, we added now the below paragraph to the methods section page 26 at lines 6-16. This method also accounts for differences in cell numbers from normal and tumor datasets.*

*“Before conducting tumor to normal comparisons, quality control was performed and patient samples with less than 200 cells (P180305N, P190125N and P180606N) were removed from the dataset (Fig S1a). In addition, for ratio calculations on myeloid cells, fibroblast/mural cells, endothelial cells, and T cells, samples with less than 50 cells of the corresponding cell type were excluded from the analysis of the corresponding cell type. To normalize for differences in the total cell count per sample we next calculated the percentage of every cell type per sample. To identify cell types whose abundance changes with malignant transformation, we then performed a Wilcoxon rank-sum test (figure S2b, S2h, S5, S10c) on samples from normal and tumor tissue. To visualize cell type abundances, we finally calculated a fold change between tumor and normal based on the average of the cell type percentages across all included patient samples.”*

Similarly, a greater discussion of inter-sample differences would be beneficial. For example, I note that batch integration methods and HSP-based gene signature regression have been used, but no discussion is made as to the cell cluster composition in terms of the number of different tumours (i.e., whether some are dominated by cells from a single/minority of tumours). I note that this has been shown visually, via UMAP in fig S1E, but some more detailed discussion and quantitation should be given to complement this. This is particularly important as the authors identify 96 cell-types, which is a large number and therefore it is useful to understand which of these are potentially found in only a small subset of tumours.

*Reply 8:*

*We fully agree that analyzing cell cluster composition is important to avoid overinterpreting clustering based on sample specific batch effects. Therefore, as advised, we have added a bar plot visualizing the number of samples contributing to each of the 81 clusters (see panel below) and corresponding text (see below) to the supplementary figures (Fig S1g) and the methods section of the manuscript at page 22 lines 22-25.*

*“Dimensionality reduction analysis applied to the expression data revealed that cells clustered primarily based on their tissue origins and subtypes but not based on patient origin. To further evaluate cluster compositions, we counted the number of patient samples contributing to each of our 81 minor cluster and found that all clusters contained samples from at least five or more patients (Fig S1g).”*

As part of the focus on non-epithelial cells in the TME, EPCAM+ cells are depleted prior to single-cell capture. However, a number of points arise from this process:

Even after depleting cells of non-epithelial origin, a substantial number of epi cells seem to remain (fig 1b). Could the authors comment on significance of these cells and whether they have been seen in other studies of gastric cancer. For instance, are these neoplastic cells or also found in the matching normal tissue? Do they express EPCAM in the single-cell data?

*Reply 9.1:*

*We address this point together with question 9.2. below.*

Also, these cells are clearly playing a role in the TME and are found to be well-connected/enriched in a number of analyses (e.g., the cell-cell communication networks) again warranting further discussion.

*Reply 9.2:*

*We thank the reviewer for this observation. We have now added an in-depth section on the epithelial cells to the manuscript where we performed clustering, annotation, copy number calling for malignant cell detection, and cell-communication analysis. Please find the new section on epithelial cells below, as well as from page 12 line 6 to page 13 line 5 in the manuscript. We hope these additions address both question 9.1 and 9.2.*

*“Top ligand-receptor interactions between gastric malignant cells and tumor microenvironment suggests mechanisms of immune evasion*

*Although we depleted EPCAM+ cells before single cell sequencing to enrich for cells of the TME, our dataset also contains 7,210 epithelial cells. These cells clustered into fifteen groups (Fig. S10a) which we annotated as gland mucus cells (GMCs) (MUC6 and TFF2), pit mucus cells (PMCs) (MUC5AC and TFF1), parietal cells (ATP4A and ATP4B), chief cells (PGA3 and PGA4), enterocytes (FABP1 and APOA1), as well as malignant cells (Fig S10a-b). The latter were identified via the copyKAT algorithm (Fig S10d) that quantifies the level of aneuploid chromosomal regions in cells. Malignant cells (designated EP11-MSC-CLDN7) were found only in the tumor samples (Fig S10a-b) and expressed the highest levels of EPCAM (Fig S10e) although more sporadic expression of this marker was also seen in the other epithelial cell clusters including enterocytes and GMCs, indicating that our EPCAM cell depletion has been incomplete. High EPCAM levels in EP11 correlated positively with the presence of cell cycle markers in this cluster further confirming the malignant nature of these cells. (Fig S10e).*

*Malignant cell cluster EP11-CLDN7 was connected to 33 cell types in the cell-interaction network (Fig 6A). The top interacting cell types were thereby CD8+ T cells as well as neutrophiles and F13-CTHRC1 fibroblasts (Fig S10f). The interaction between EP11 and CD8+ T cells was dominated by the interactions of LGALS9 – HAVCR2 and HVEM – CD160 both of which are well-known checkpoints that inhibit CD8+ T cell functionality (Fig S10g), suggesting an immunosuppressive function of the gastric tumor cells. EP11 and F13 cells are predicted to interact through FZD5 – WNT5A, reinforcing the hypothesis of F13 being a fibroblast cluster that is potentially facilitating tumor cell migration 21. Lastly, neutrophils were connected to EP11 cells via chemokine-receptor pairs CXCL1/2/3/5/8 – CXCR1/2. This could be an important axis for the attraction of neutrophils which have been described as immunosuppressive in gastric cancer 22,23.”*

I wonder what effect the depletion of EPCAM+ epithelial cells has on the biclustering based approach and resultant identification of response and non-response groups (e.g., as shown in fig 8 and S9).

*Reply 9.3.1:*

*We thank the reviewer for this very valid question. Our bi-clustering algorithm takes a list of differentially expressed genes from bulk data (which contains all cell types) and clusters them based on their expression profiles in the single cell data. As such, the algorithm requires each cell type involved in the observed differential expression from the bulk samples to also be present in the single cell data. Results are thereby highly robust against the number of cells in each single cell cluster as long as the transcription profile of the cell type is sufficiently represented in the data. As shown above (reply 9.1 and 9.2), our dataset does contain also EPCAM positive cells and a rather comprehensive list of epithelial cell types including CLDN7 positive tumor cells.*

*To assess the robustness of the bi-clustering method to changes in cell frequencies (as might arise from depleting or enriching cell types or after IO treatment), we compared the gene clusters obtained from the original data to clusters arising from downsampled versions of our single cell dataset where we randomly remove 90% of the fibroblasts before re-running the bi-clustering algorithm. As shown exemplary below in panel A, the majority of clusters are not affected by the downsampling of the dataset and all, but three original clusters have a well matching counterpart in the downsampled data, sharing most of the assigned genes. Repeating this analysis 100 times revealed that all clusters preserved at least 50% of the originally assigned genes (see panel B below). Further, to evaluate if the conclusions drawn from subsequent gene set enrichment analysis were also stable against changes in cell type frequencies, we tracked the ranks of the top 10 GO ontology terms enriched in original cluster U7 (genes in this cluster were highly expressed by F13-Act-CTHRC1), across each of the 100 downsampled versions and found that the ranks of these terms were highly conserved (see panel C below). Based on these observations we believe that the bi-clustering results are largely unaffected by our TME enrichment approach. We decided to add the downsampling example on fibroblasts as supplementary figure S4f-h and describe the above procedure in the methods section of the manuscript at page 23 lines 9-13 and shown below.*

*“To assess the robustness of the results obtained by the bi-clustering approach against changes in cell frequencies in the single cell data, we applied the algorithm to 100 downsampled versions of our dataset where each time 90% of the fibroblast cells had been randomly selected and removed. Results obtained from the original and downsampled data were found to be highly similar with almost all clusters preserving at least 50% of the genes that were originally assigned to them (Fig S4f-h).”*

For instance cluster R4 is strongly enriched for epithelial properties. Could the authors comment on this?

*Reply 9.3.2:*

*R4 as well as NR3 and NR8 are indeed enriched with epithelial specific genes (Fig 8b and S12a-b). This is confirmed by comparing the genes assigned to these clusters by the bi-clustering algorithm to the epithelial marker genes published by Zhang et al. 24, NR3 and NR8 thereby appear to be most specific for pit mucus cells and malignant cells, respectively, while R4 is enriched for pan-epithelial markers (see panels A and C below). We hypothesize that the reason why we find epithelial gene signatures to be differentially expressed between responders and non-responders is that non-responders seem to have an increased number of pit mucus and malignant cells as shown by the differential expression of AKR1C2 and KRT20, respectively, while responders tend to have a higher overall number of epithelial cells as indicated by the expression of pan-epithelial cell specific markers KRT8 and KRT18 (panels D-F).*

I think that the term "comprehensive" (conclusion, line 29) is not quite correct for this study as the representation of the epithelial cell compartment is incomplete due to EPCAM+ cell depletion. I suggest re-phrasing this.

*Reply 9.4:*

*As mentioned above, the data set still contains a variety of EPCAM positive cells including malignant cells with high EPCAM expression levels. Nevertheless, as pointed out by the reviewer, we cannot completely exclude the possibility of missing certain epithelial cell types with very high EPCAM expression. We therefore now rephrased the sentence as follows:*

*“Our gastric cancer single cell TME compendium together with the matched bulk transcriptome data provides a unique resource for the identification of new potential biomarkers for patient stratification and helps further elucidate gastric cancer biology and insights for therapy.”*

Also, (on pg 12, lines 52-53) when stating that "non-responder gene programs were associated with stromal, plasma, and epithelial cells …" it's unclear which epithelial cells are being referred to here, i.e., are these the EPCAM- cells in the single-cell dataset? I think this needs clarifying.

*REPLY 9.5: We thank the reviewer for pointing out this ambiguity.*

*More extensive heatmaps with detailed clusters information have now been added (Fig S12A-B). As mentioned above, non-responder gene cluster 3 (NR3) corresponds to pit mucus cells and non-responder gene cluster 8 (NR8) corresponds to EPCAM+ malignant cells (EP11-Malignant-CLDN7). We have changed the sentence at page 18 line 23-25 in the manuscript to clarify this issue. Below please find the updated sentence.*

*“Furthermore, non-responder gene programs were associated with stromal, plasma, and EPCAM-negative PMCs as well as EPCAM-positive malignant epithelial cells and highlight potential drug targets for patient selection or therapeutic intervention.”*

As part of the cell-communication network analysis the authors note that "communication models in tumour and normal tissue differed significantly (Fig. S6)". This is not immediately clear to me. I think a more quantitative justification for this conclusion is needed. Also, the colours of the cell-types are different in the rows and columns of the matrix shown in this figure. This is confusing and I think needs a clearer figure legend.

*Reply 10:*

*We thank the reviewer for this suggestion. We have now introduced a quantitative comparison of the cell communication models by evaluating the difference in the closeness centrality, which represents how central a cell type is to the network, between the networks derived from normal and tumor tissue. We have added the top cell types by closeness centrality to supplementary figure S8c-d and added the following sentence to the main text at page 11 line 11-12: “Communication models derived from the cells of the TME and from normal gastric tissue thereby differed significantly in their structure, with different cell subtypes ranked most central in the interaction networks for TME and normal tissue, and F13-CTHRC1 as a central network hub in the TME but not in normal tissue (Fig. S8c, S8d).” We also revised the annotation color in the heatmap to be consistent in rows, columns, and the new corresponding closeness centrality bar plot (Fig. S8).*

The authors use the cell-types identified in the TME to generate binary classifiers to predict contribution to ICI response. This is done in the form of a series of AUROCs. However, it's difficult to appreciate the significance/impact of the difference between, for example, an AUROC of 0.85 (cycling CD8+ T-cells) and 0.81 (regular CD8+ T-cells). Is there any way to further quantify these differences? Further, it would be useful to see the full list of AUROC scores for all 96 cell-types as a supplemental figure or table to get an appreciation of the variability of performance for each celltype.

*Reply 11: We fully agree with the reviewer that the differences between the cycling and regular T cells is not striking. We have revised the text in the manuscript to not focus on this difference but just to highlight which cell types best correlate with response. Nevertheless, to further evaluate the differences between the survival curves obtained with the different signatures we have now also added confusion matrices as well as performance measurements of the T27 cycling CD8 T cells signature, CD8 T cell signature, F14 fibroblast signature, and the fibroblast signature as supplementary figure S12h-i to the manuscript. As shown also below in panels A and C, these results confirm that the T27 cycling CD8 T cell signature represents an improvement over the pan-CD8 T cell signature in terms of increased accuracy, positive predictive value (PPV), and specificity while maintaining a similar sensitivity (panel A and C below). The F14 signature shows an improved PPV and perfect specificity compared to the pan-fibroblast signature at the expense of a slight reduction in sensitivity (see panels B and C below). We have added performance metrics on all 81 cell types to supplementary table S6 and changed the text from page 15 line 22 to page 16 line 2 to:*

*“With an area under the ROC curve of 0.85, the presence of T27-cycling CD8+ T cells followed by the presence of pan CD8 T cells (AUROC of 0.81) was found to best predict treatment outcome in this cohort (Fig. 8e). Presence of F14-ADAM28 activated fibroblasts followed by presence of pan-fibroblasts were best predictive for non-response with AUROCs of 0.82 and 0.7, respectively (Fig. 8e) and up to 100% specificity (Fig. S12h-i and supplementary table S6).”*

Data processing and availability

Pg14 line 5: notes that 117506 "post-filter" cells were identified, however, in the results on pg5 only 96,623 cells were used in the clustering. I assume this is related to the "low-quality" high mitochondrial cluster that was removed (pg 14/Fig S1d)? Could the authors please clarify this.

*Reply 12:*

*As expected by the reviewer, the difference in cell number is indeed due to the "low-quality” high mitochondrial cluster that we mention in Fig S1c-d. We now included the exact number of post-filter cells in the methods section at page 21 line 5 and line 25.*

This will be a valuable resource to the community and therefore it's vital that data and analysis code be made available upon publication. As a minimum all processed results and analysis scripts should be made publicly available (e.g., in GEO and github), with raw data available through appropriate access-controlled channels. I also suggest adding the single-cell data to a repository such as the Broad single-cell explorer. These steps will ensure reproducibility and much increase the audience and accessibility of the resulting dataset.

*Reply 13:*

*We fully agree with the reviewer’s suggestion and have now uploaded the data and analysis code to public domain for reproducibility and accessibility. The processed data is deposited to GEO (GSE206785) with raw sequence available in GSA (HRA002336). The analysis code is available on Github (Repo chriscainx/gastric-cancer).*

Minor comments

Pg6, lines 54-56: "In addition, the RNA expression of these genes is upregulated in tumors from many different indications in the cancer genome atlas (TCGA) cohort (Fig S2d).". This figure only shows the gene CTHRC1, whereas the text indicates that it should show more genes?

*Reply 14:*

*As pointed out by the reviewer, we only plot the expression of CTHRC1. This has now been clarified in the text (page 6 line 6-7).*

Pg7, lines 9-10: "Upon analyzing the 3,684 endothelial cells in our data, we found eight clusters that corresponded to six different endothelial cell subtypes (Fig. 3a).". The cluster labels are difficult to read, but I think that there are actually 9 EN cluster shown? Also, the numbering is confusing as the cluster labels indicate that there should be at least 12 clusters (i.e., EN01-EN12)? This should be clarified.

*Reply 15:*

*We thank the reviewer for catching this typo. There are indeed 9 endothelial cell clusters. This has now been corrected in the manuscript. The detailed analysis on the endothelial cells necessitated and additional QC step with the removal of some low-quality endothelial clusters: EN08, EN09, EN11 and EN13. We added an additional paragraph to the methods section at page 22 line 10-13 to address the removal of these clusters.*

Fig 3c: It's not clear what the criteria for selecting this particular set of genes was.

*Reply 16:*

*Genes shown in Fig 3c represent the intersect of the top differentially expressed genes between tumor endothelial cells (EN03 and EN10) and the endothelial cells enriched in normal tissue (full list is available in Table S3) and the genes associated with the most significantly enriched gene ontology terms. We now described the procedure on how these genes were selected in the main text at page 8 line 1-6 and in the legend of figure 3.*

I think that "domestic" should be replaced with "tissue resident" in the conclusion (line 25)?

*Reply 17:*

*This has now been changed in the text at page 18 line 3.*

Reviewer #3

*Reply: We thank the reviewer for the positive assessment of the quality and impact of our work and very much welcome the constructive recommendations for changes which we have addressed point by point below.*

Major comments

Some markers used in this study such as RGS5 mark pericytes as well - have the authors ensured exclusion of this cell type in the fibroblast cluster?

*Reply 18.1:*

*We would like to thank the reviewer for this insightful comment which helped us improve our annotation. As shown in Fig 1 in the manuscript we found three clearly distinct clusters of stromal related cells in our data set. One of these clusters contained highly expressed glial cell markers: SOX10, CDH19 and PLP1 which we therefore continue to label as glial cells. The other two clusters were labeled as fibroblasts based on the expression of COL1A1, PDGFRA and ACTA2. After taking your comment into consideration, we now changed our annotation for one of the clusters from myofibroblast to mural cells 25 which comprise both pericytes and vascular smooth muscle cells. These cells are negative for canonical fibroblast markers such as LUM, DCN and express only lower levels of collagens (see panels A-B below). The corresponding subcluster F07, F11, and F12 were now renamed in the manuscript to L07-SMC-RERGL, L11-Pericyte-MT1A, L12-Pericyte-ANGTP2 and are designated as mural cells. We updated the annotation across all figures and in the text of the manuscript. The third stromal cell related cluster we kept annotated as fibroblasts due to the expression of DCN, LUM and COL1A1 and the similarity of these cells to an atlas of perturbed human fibroblast from NSCLC and PDAC fibroblasts recently published by Buechler et al. 26 (see panel C below). The changes in the manuscript are found from page 5 line 15 to page 6 line 12.*

Can the authors provide references and/or reasoning for categorizing fibroblasts into: resting, activated and myofibroblasts?

*Reply 18.2:*

*Fibroblasts were annotated into activated and resting fibroblasts based on markers found in the literature that match the marker genes of our two visually well separated clusters (F08, F13, and F14 versus F01, F02, F03, F05, F06, F09 and F10). Resting fibroblast are marked by the expression of S100A4, CFD, DPT, FBLN1 25–28 (panel A below). The other main fibroblast cluster contained broad expression of CXCL14, POSTN, APOE, COL3A1 and FAP, genes linked to fibroblast activation 26–28 (panel A).*

*We compared gene signatures as defined by Buechler et al. 26 to our clusters and observed a clear similarity of F01, F02, F03, F09 and F10 with PI16+ adventitial fibroblasts; F14, F05 and F06 with NPNT+ fibroblasts; and F13 with LRRC15+ and COL3A1+ myofibroblasts (see panel C below). F08 fibroblasts appear unique for the gastric stroma as they don’t express any of the fibroblast signatures published by Buechler et al. Lastly, F12, which we have now re-annotated as pericyte in the manuscript, also display some fibroblast-like characteristics such as the expression of COL3A1+ while in addition expressing pericyte specific genes such as MYH9, RGS5 and CSPG4 (C). We have added our reasoning for the annotation together with references on resting and activated fibroblast to the main text from page 5 line 15 to page 6 line 12.*

Fibroblast clusters described (for e.g., resting fibroblasts) are dictated by the choice of resolution parameters used for clustering. Can the authors comment why so many different resting fibroblasts are observed in normal adjacent tissue samples - are these distinct cell types or cell states? Applying algorithms such as cNMF [Kotliar et al., 2019 eLife] will help clarify this.

*Reply 19:*

*As suggested by the Reviewer, we performed cNMF analysis on all fibroblast populations. Among all cells of the 10 fibroblast subsets, we identified 11 gene expression modules. Most of the fibroblast subtypes show a specific high expression of one of these 11 modules. For example, F01 specifically expresses module 10, and F13 specifically expresses module 3. The only exceptions are F05, F06, F02, and F10. F05 and F06 have similar usage of gene module 1, whereas F02 and F10 have similar usage of gene module 9. However, when directly comparing these cell types (see Figure 2 in the manuscript), we found F05 and F06 to differentially express genes such as THBS4 and TAC1, while F02 and F10 showed different expression levels of genes such as IGFBP6 and CFD. We therefore conclude that F05/F06 and F02/F10 are possibly related cell states, but because they expressed different genes, we suggest keeping the original unsupervised partitioning of these cell subclusters and added a description of the above fibroblast state analysis in Results section of the main text at page 5 lines 24-26, and in supplementary figure (Figure S3).*

What are the differences in genes between U1 and U6, both implicated in ECM organization? In Figure 2C F13-CTHRC1 upregulates genes in U1 and U7 involving processes of ECM organization and destruction - can the authors explain the functional role of CTHRC1 fibroblasts given that they express genes from opposing processes?

*Reply 20.1:*

*Our bi-clustering approach groups genes according to their expression across the cell types in the single cell data. Cluster U1 is shared among resting and activated fibroblasts and contains genes that are involved in collagen biosynthesis and extracellular matrix (ECM) formation such as COL1A1, COL1A2, COL6A3, COL12A1, THBS1, LUM and FN1. In contrast, genes of cluster U6 are expressed mostly by F12 and can be grouped into playing a role in ECM organization (e.g., ACAN, COL5A3, BGN, NID2 and IBSP) or as being important for angiogenesis (e.g., PGF, ANGPT2, EGFL6 and PDGFRB). Genes of cluster U7 are specifically expressed in F13 myofibroblasts and mostly contain metalloproteinases such as MMP1, MMP3, MMP13; cytokines such as CXCL6, IL11, TNFSF11; and migration related genes such as WNT5A and PDPN. It appears not uncommon for CAFs to function both as ECM producers as ECM destructors as this process is crucial for matrix remodeling and invasion of the tumor cells 30. We have adapted figure 2C and the main text from page 6 line 22 to page 7 line 5 to clarify the differences.*

Can the authors classify various fibroblast subsets based on the ECM atlas described here: matrisome project

*Reply 20.2:*

*We thank the reviewer for pointing us to this valuable resource. We performed multiple queries of gene signatures from this resource across our fibroblast subsets. Although many genes are not differentially expressed or not detected in our data, the fibroblast subsets display very distinct profiles in all six categories of extracellular matrix-related genes, highlighting their unique functions in shaping the extracellular matrix. We included this result in the supplementary figure (Fig S3) and added a description in the main text at page 6 lines 1-3.*

In Figure 8D, it will be important to understand which resting fibroblast populations express high NR11 genes (given that this signature is restricted to resting fibroblasts)

*Reply 21:*

*As suggested by the reviewer, we have looked more in detail at signature NR11. It is highly expressed in F01 and F02 fibroblasts compared to other resting fibroblasts (panel below). As we do not explicitly know the impact of gene signature NR11 we would suggest to not add this information to the main text.*

Do F13-CTHRC1 express LRRC15 and is this population driven by TGF-beta signaling as well?

*Reply 22.1:*

*In our dataset, we observed a higher expression of TGFB1 in F13 compared to the other fibroblast populations. Interestingly, while LRRC15 is only sporadically detected in fibroblasts from gastric cancer, we find the cells from F13 to have the highest level of expression of this gene. However, as we cannot conclude based on our data whether TGFB is driving LRRC15 expression or whether it is driving differentiation of the F13 subtype in an autocrine fashion we would suggest to not include this hypothesis in the text.*

Can the authors comment on why F14-ADAM28+ fibroblasts are more predictive of response versus non-response in the STAD cohort as opposed to F13-CTHRC1 that interact with multiple cell types in the GC single-cell atlas?

*Reply 22.2:*

*We would like to thank the reviewer for this comment. It is known that the gastric cancer molecular subtypes MSI-high and EBV respond well to immunotherapy treatment while CIN and GS mostly do not respond 31. Differential expression of our signature among these cancer subtypes might therefore provide a possible explanation for its predictive power. Hence, we investigated the expression of our F14 and F13 signatures in the molecular subtypes of the STAD cohort from TCGA. In contrast to the F13 signature, we observe a significant increase of the F14 signature in GS patients (see panels A-B below). This suggests that our F14 signature can be potentially used as an additional biomarker for identifying GS patients. We want to highlight that the F14 signature also contains a number of genes with potential immunosuppressive function, such as APOE32 and CCL11 which increases the proportion of Tregs in the tumor 33. Nevertheless, the exact role of this fibroblast subtype in GS and perhaps also other patients remains elusive. In the revised manuscript, we added the below panel to Fig S12j-k and the following sentence at page 16 lines 21-23 to the discussion describing this observation.*

*“However, we found the marker gene signature from F14-ADAM28 fibroblasts to be differentially expressed in genomically stable patients (Fig S12j-k), who in general don’t respond to immunotherapy treatment.”*

Have the authors considered categorizing impactful subpopulations within each cell type by tumor mutational burden or molecular subtypes - is a subpopulation overrepresented in one subtype over another?

*Reply 23:*

*We compared the cell subtype composition of the different molecular subtypes present in our patient cohort as well as with the tumor mutation burden in each tumor. While we did not find any significant association between subpopulation frequencies and tumor mutation burden, we identified a fibroblast subpopulation that appears enriched in specific molecular subtypes (as mentioned above in Reply 22.2). We have added this observation to the manuscript in the discussion at page 16 lines 20-23.*

Can the authors show survival outcomes in the TCGA cohort for non-inflammatory and proinflammatory macrophage subpopulations enriched in GC as opposed to a common pro and non-inflammatory gene signature?

*Reply 24:*

*As requested by the Reviewer, we performed a comprehensive survival analysis for the mentioned macrophage subsets. Although the deconvolution results for these cell types are likely accurate (as shown in Fig S14 and in reply 9.3), we did not find a significant survival difference associated with these cell types. We believe this is not surprising as the pro- and anti-inflammatory gene signatures are not only expressed by macrophages but across several immune cell types (panel below). Limiting the analysis to macrophages therefore likely weakens the total gene expression signal. We added the following sentence to the manuscript at page 9 line 18-20 to clarify this situation:*

*“However, the abundance of the corresponding macrophage populations as determined by CIBERSORTx deconvolution of the bulk RNA-seq samples was not predictive for outcome (data not shown)”.*

Sathe et al., 2020 Clinical Cancer Res  also describe a single cell atlas of the gastric cancer TME, albeit with fewer patients. Do the authors also observe the LGR4-RSPO3 axis in fibroblasts and Notch signaling characteristic of endothelial cells?

*REPLY 25.1: We thank the reviewer for highlighting this finding. We have now performed the same analysis as in Sathe et al. on our dataset and indeed observe a similar expression pattern of Wnt-signalling components such as RSPO3 in fibroblasts and preferential expression of Notch signaling components in endothelial cells (compare panels A and B with C and D below). In the manuscript we have now added one sentence to the Discussion from page 16 line 26 to page 17 line 2 and added a supplementary figure to point out this consistent finding (Fig S13).*

It will be worthwhile to map/project this dataset on your atlas and update current knowledge about the gastric TME ecosystem.

*REPLY 25.2: We performed the mapping of Sathe dataset to our single cell atlas and have created a notebook page titled “Integrating with other gastric cancer atlas” which we uploaded to our Github repository for public access.*

Minor comments

Figure 2A: Use contrasting colors to represent tumor and non-tumor samples. It is hard to distinguish between two similar shades of green.

*REPLY 26: As requested by the reviewer this has now been changed. We have switched the panels of Fig 2a, 3a, 4a, 5b, S1e, S2b, S2h, S5, S6a-b, S10a, S10c.*

Figure 2C: Please clearly mention in the legend or annotate in the figure that U1, D9 etc correspond to gene sets up- or down-regulated in bulk tumor versus normal comparison.

*REPLY 27: As suggested by the reviewer, this information has now been added to the legend of figure 2.*

Figure 5: Can the authors show tumor versus normal UMAP here for the readers to quickly assess where the different T cell populations are localized?

*REPLY 28: We have now added two extra panels to figure 5 visualizing normal and tumor CD4 and CD8 T cells for easy assessment of their UMAP projections.*

Figure 8A: Increase font size of gene names.

*REPLY 29: As requested by the reviewer, the size of the gene names in Fig 8A has been increased.*

Figure 7 survival plots: add risk table clearly showing number of patients per group (survminer package in R)

*REPLY 30: As requested, we have added risk tables to all survival curves in Figure 7.*

Figure S9D and E: Please check whether the Pearson correlation is computed for panel D or E (average NR and R score per patient or NR13 versus R5. Presumably, this is for E). Could the authors also add a legend for molecular subtypes for D and E (in addition to the one in C)?

*REPLY 31: We thank the reviewer for pointing out the missing legend and description. We have added legends now to panels D and E. We also added to both plots the corresponding Pearson correlation coefficients. In contrast to panel E no negative correlation is observed in panel D.*

Please use consistent nomenclature: anti-inflammatory macrophage as opposed to non-inflammatory.

*REPLY 32: as requested by the reviewer the term non-inflammatory macrophages has been changed to anti-inflammatory macrophages in the text and figures.*

Grammar: check for usage of where and were. E.g. page 11, line 60: "Activated endothelial subtypes have been documented in prostate cancer and 'where' shown to express CAF markers". 'Where' should be replaced with "were".

*REPLY 33: We thank the review for catching this error.*

*References*

*1. Yost, K. E. et al. Clonal replacement of tumor-specific T cells following PD-1 blockade. Nat. Med. 25, 1251–1259 (2019).*

*2. Lee, A. H. et al. Neoadjuvant PD-1 blockade induces T cell and cDC1 activation but fails to overcome the immunosuppressive tumor associated macrophages in recurrent glioblastoma. Nat. Commun. 12, 6938 (2021).*

*3. Bassez, A. et al. A single-cell map of intratumoral changes during anti-PD1 treatment of patients with breast cancer. Nat. Med. 27, 820–832 (2021).*

*4. Avila Cobos, F., Alquicira-Hernandez, J., Powell, J. E., Mestdagh, P. & De Preter, K. Benchmarking of cell type deconvolution pipelines for transcriptomics data. Nat. Commun. 11, 5650 (2020).*

*5. Goveia, J. et al. An Integrated Gene Expression Landscape Profiling Approach to Identify Lung Tumor Endothelial Cell Heterogeneity and Angiogenic Candidates. Cancer Cell 37, 21-36.e13 (2020).*

*6. Takayama, Y. et al. Inhibition of PAI-1 Limits Tumor Angiogenesis Regardless of Angiogenic Stimuli in Malignant Pleural Mesothelioma. Cancer Res. 76, 3285–3294 (2016).*

*7. Chen, S. et al. SERPINE1 Overexpression Promotes Malignant Progression and Poor Prognosis of Gastric Cancer. J. Oncol. 2022, e2647825 (2022).*

*8. Oshi, M. et al. Angiogenesis is associated with an attenuated tumor microenvironment, aggressive biology, and worse survival in gastric cancer patients. Am. J. Cancer Res. 11, 1659–1671 (2021).*

*9. Baitsch, D. et al. APOLIPOPROTEIN E (APOE) INDUCES ANTI-INFLAMMATORY PHENOTYPE IN MACROPHAGES. Arterioscler. Thromb. Vasc. Biol. 31, 1160–1168 (2011).*

*10. Kim, H. The transcription factor MafB promotes anti-inflammatory M2 polarization and cholesterol efflux in macrophages. Sci. Rep. 7, 7591 (2017).*

*11. Revel, M., Sautès-Fridman, C., Fridman, W.-H. & Roumenina, L. T. C1q+ macrophages: passengers or drivers of cancer progression. Trends Cancer 0, (2022).*

*12. Ramos, R. N. et al. Tissue-resident FOLR2+ macrophages associate with CD8+ T cell infiltration in human breast cancer. Cell 185, 1189-1207.e25 (2022).*

*13. Viola, M. F. & Boeckxstaens, G. Intestinal resident macrophages: Multitaskers of the gut. Neurogastroenterol. Motil. 32, e13843 (2020).*

*14. Wang, Y. et al. Tissue-resident macrophages promote extracellular matrix homeostasis in the mammary gland stroma of nulliparous mice. eLife 9, e57438 (2020).*

*15. Barrett, C. W. et al. Selenoprotein P influences colitis-induced tumorigenesis by mediating stemness and oxidative damage. J. Clin. Invest. 125, 2646–2660 (2015).*

*16. Beckers, C. M. L. et al. Cre/lox Studies Identify Resident Macrophages as the Major Source of Circulating Coagulation Factor XIII-A. Arterioscler. Thromb. Vasc. Biol. 37, 1494–1502 (2017).*

*17. Lambrechts, D. et al. Phenotype molding of stromal cells in the lung tumor microenvironment. Nat. Med. 24, 1277–1289 (2018).*

*18. Zhang, Y. et al. Single-cell analyses reveal key immune cell subsets associated with response to PD-L1 blockade in triple-negative breast cancer. Cancer Cell 39, 1578-1593.e8 (2021).*

*19. Zhang, Q. et al. Landscape and Dynamics of Single Immune Cells in Hepatocellular Carcinoma. Cell 179, 829-845.e20 (2019).*

*20. Zhang, L. et al. Single-Cell Analyses Inform Mechanisms of Myeloid-Targeted Therapies in Colon Cancer. Cell 181, 442-459.e29 (2020).*

*21. Weeraratna, A. T. et al. Wnt5a signaling directly affects cell motility and invasion of metastatic melanoma. Cancer Cell 1, 279–288 (2002).*

*22. Wang, T. et al. Tumour-activated neutrophils in gastric cancer foster immune suppression and disease progression through GM-CSF-PD-L1 pathway. Gut 66, 1900–1911 (2017).*

*23. Zhou, Z. et al. A C-X-C Chemokine Receptor Type 2–Dominated Cross-talk between Tumor Cells and Macrophages Drives Gastric Cancer Metastasis. Clin. Cancer Res. 25, 3317–3328 (2019).*

*24. Zhang, P. et al. Dissecting the Single-Cell Transcriptome Network Underlying Gastric Premalignant Lesions and Early Gastric Cancer. Cell Rep. 27, 1934-1947.e5 (2019).*

*25. Muhl, L. et al. Single-cell analysis uncovers fibroblast heterogeneity and criteria for fibroblast and mural cell identification and discrimination. Nat. Commun. 11, 3953 (2020).*

*26. Buechler, M. B. et al. Cross-tissue organization of the fibroblast lineage. Nature 593, 575–579 (2021).*

*27. Kalluri, R. The biology and function of fibroblasts in cancer. Nat. Rev. Cancer 16, 582–598 (2016).*

*28. Sahai, E. et al. A framework for advancing our understanding of cancer-associated fibroblasts. Nat. Rev. Cancer 20, 174–186 (2020).*

*29. Lee, H.-O. et al. Lineage-dependent gene expression programs influence the immune landscape of colorectal cancer. Nat. Genet. 52, 594–603 (2020).*

*30. Winkler, J., Abisoye-Ogunniyan, A., Metcalf, K. J. & Werb, Z. Concepts of extracellular matrix remodelling in tumour progression and metastasis. Nat. Commun. 11, 5120 (2020).*

*31. Kim, S. T. et al. Comprehensive molecular characterization of clinical responses to PD-1 inhibition in metastatic gastric cancer. Nat. Med. 24, 1449–1458 (2018).*

*32. Kemp, S. B. et al. Apolipoprotein E Promotes Immune Suppression in Pancreatic Cancer through NF-κB–Mediated Production of CXCL1. Cancer Res. 81, 4305–4318 (2021).*

*33. Wang, R. & Huang, K. CCL11 increases the proportion of CD4+CD25+Foxp3+ Treg cells and the production of IL‑2 and TGF‑β by CD4+ T cells via the STAT5 signaling pathway. Mol. Med. Rep. 21, 2522–2532 (2020).*

*34. Sathe, A. et al. Single-Cell Genomic Characterization Reveals the Cellular Reprogramming of the Gastric Tumor Microenvironment. Clin. Cancer Res. 26, 2640–2653 (2020).*

**Second round of review**

**Reviewer 2**

All of my comments have been addressed. Thank you for your detailed and thoughtful responses to my questions.

**Reviewer 4**

Major concerns:

1. Page 6, line3-5. Why F13-CTHRC1 was selected as the highly cancer associated fibroblasts? In Fig. 2B, there seems no significant difference between tumor and normal tissue for F13. In contrast, F14 is significantly higher in tumor than normal tissue. However, in Fig. S2b, F13 got significant difference while F14 not did not.

2. Page 8, line 11-19. The authors found that “SERPINE1 expression was associated with a drastically worsened survival in the independent gastric cancer cohort from TCGA”, but the final conclusion “EN10-SERPINE1 endothelial cells could be targeted to block the metastatic spread of gastric tumors” is not solid --- could the authors check if other EN10 specific genes were also significantly related with worsened survival? There exists a possibility that some other genes could be associated with better survival of gastric cancer.

3. Page 9, line9-11. the authors claimed that “the gastric TME was characterized by a substantial increase in M08-CXCL5 proinflammatory as well as M07-APOE”, while M08-CXCL5 ratio change between tumor and normal tissue seems not significant in Fig4C.

4. Page11, line15-26: F13-CTHRC1 communicates with endothelial cells, macrophages, T cells and DCs. Is there other cells types that does not communicate with F13-CTHRC1? If yes, please show them with figures similar to Fig6C and Fig6D.

5. Per the Reviewer2’s concern ‘I wonder what effect the depletion of EPCAM+ epithelial cells have on the biclustering based approach and resultant identification of response and non-response groups (e.g., as shown in fig 8 and S9)’, the authors’ response does not perform analysis between response and non-response group, thus Reply9.3.1 and 9.3.2 is incomplete.

6. The Reply 2 referred to a new figure, which is not shown. It would be good to include it as a supplementary figure and add corresponding text in the main text.

Minor questions:

1. p values in Fig. 5C are log10-transformed, while p values in Fig. S14A are loge-transformed. They should be consistent.

2. Page 11, line24-26. There is no TSLP-TSLPR in Fig. S9d.

**Authors Response**

**Point-by-point responses to the reviewers’ comments:**

Reviewer #4:

Major concerns:

1. Page 6, line3-5. Why F13-CTHRC1 was selected as the highly cancer associated fibroblasts? In Fig. 2B, there seems no significant difference between tumor and normal tissue for F13. In contrast, F14 is significantly higher in tumor than normal tissue. However, in Fig. S2b, F13 got significant difference while F14 not did not.

*Reply 1: We would like to thank the reviewer for noticing the mismatch in annotations that occurred when redrawing the figure for resubmission. The ordering has now been corrected.*

2. Page 8, line 11-19. The authors found that “SERPINE1 expression was associated with a drastically worsened survival in the independent gastric cancer cohort from TCGA”, but the final conclusion “EN10-SERPINE1 endothelial cells could be targeted to block the metastatic spread of gastric tumors” is not solid --- could the authors check if other EN10 specific genes were also significantly related with worsened survival? There exists a possibility that some other genes could be associated with better survival of gastric cancer.

*Reply 2: As suggested by the reviewer we have redone the survival analysis with the top 20 marker genes of EN10 endothelial cells which shows a similar pattern with worsened survival for the patient group expressing the high EN10 gene signature (p = 0.039, HR = 1.4). We have added this plot as figure panel 3E and the corresponding sentence to the main text.*

3. Page 9, line9-11. the authors claimed that “the gastric TME was characterized by a substantial increase in M08-CXCL5 proinflammatory as well as M07-APOE”, while M08-CXCL5 ratio change between tumor and normal tissue seems not significant in Fig4C.

*Reply 3: We agree with the reviewer and took out M08-CXCL5 myeloid cells from the list of significantly increased clusters in the gastric tumor microenvironment.*

4. Page11, line15-26: F13-CTHRC1 communicates with endothelial cells, macrophages, T cells and DCs. Is there other cells types that does not communicate with F13-CTHRC1? If yes, please show them with figures similar to Fig6C and Fig6D.

*Reply 4: As shown in Fig. 6B and Fig. 6E, with the PAGA connectivity threshold of 0.05, almost all B cell and plasma cell subclusters and some myeloid and T cell subclusters don’t significantly communicate with F13-CTHRC1 cells. Since Fig. 6C and Fig. 6D contain only the significant cell types on the y-axis, the plot of the same format for F13-CTHRC1 and the cell types they don’t communicate with will be empty. Therefore, we would suggest not to show empty plots for the non-interacting cell types.*

5. Per the Reviewer2’s concern ‘I wonder what effect the depletion of EPCAM+ epithelial cells have on the biclustering based approach and resultant identification of response and non-response groups (e.g., as shown in fig 8 and S9)’, the authors’ response does not perform analysis between response and non-response group, thus Reply9.3.1 and 9.3.2 is incomplete.

*Reply 5: Based on the question, we wonder whether there might have been a misunderstanding. The sets of response genes and non-response genes were not derived from our single cell data but from a standard differential gene expression analysis using the bulk RNA-seq samples from pembrolizumab sensitive and pembrolizumab resistant tumors as provided by Kim, S. T. et al. Nat. Med. 24, 1449–1458 (2018). The assignment of genes to either response genes or non-response genes is thus not dependent on our data and the cell types covered therein. No further comparison between the response and non-response genes was performed in our study. Instead, we investigated which cell types are the likely source of these differentially expressed genes. As shown in Figures 9.3.1 A and B the subsequent assignment of the response and non-response genes to certain cell types in our data (and corresponding gene set enrichment analyses) is in fact very robust against shallow coverage of individual cell types.*

6. The Reply 2 referred to a new figure, which is not shown. It would be good to include it as a supplementary figure and add corresponding text in the main text.

*Reply 6: The figure in reply 2 is a copy of figure panel 6A, as such the figure is already part of the main manuscript and was merely added to reply 2 for clarification.*

Minor questions:

1. p values in Fig. 5C are log10-transformed, while p values in Fig. S14A are loge-transformed. They should be consistent.

*Perhaps there was a misunderstanding as we are not showing any log-transformed p-values in our manuscript. The numbers shown in figure 5C and S14A are two different number formats, to be consistent we have changed the format in figure 5C similar to fig S14A.*

2. Page 11, line24-26. There is no TSLP-TSLPR in Fig. S9d.

*We apologize for this error. The TSLPR and IL-7Rα form a heterodimer to bind with TSLP as a receptor, and the existence of IL-7Rα is obligatory for TSLP/TSLPR signaling (Linda S. Park, Unja Martin, Kirsten Garka, Brian Gliniak, James P. Di Santo, Werner Muller, David A. Largaespada, Neal G. Copeland, Nancy A. Jenkins, Andrew G. Farr, Steven F. Ziegler, Philip J. Morrissey, Raymond Paxton, John E. Sims; Cloning of the Murine Thymic Stromal Lymphopoietin (Tslp) Receptor: Formation of a Functional Heteromeric Complex Requires Interleukin 7 Receptor. J Exp Med 5 September 2000; 192 (5): 659–670. doi: https://doi.org/10.1084/jem.192.5.659). Therefore, in our receptor ligand model and Fig. S9d, we used the symbol TSLP-IL7R to designate the TSLP to IL-7Rα/TSLPR axis. We have changed the mixed expression in the text to “TSLP-IL7R signaling”.*

**Third round of review**

**Reviewer 4**

The comments are addressed.
